# Non‐pharmacological interventions for asthma prevention and management across the life course: Umbrella review

**DOI:** 10.1002/clt2.12344

**Published:** 2024-02-29

**Authors:** Xunliang Tong, Xinyue Zhang, Mengyuan Wang, Zijun Wang, Fawu Dong, Enying Gong, Torsten Zuberbier, Yanming Li

**Affiliations:** ^1^ Department of Pulmonary and Critical Care Medicine Beijing Hospital National Centre of Gerontology Institute of Geriatric Medicine Chinese Academy of Medical Sciences Beijing China; ^2^ Beijing Hospital National Centre of Gerontology Institute of Geriatric Medicine Chinese Academy of Medical Sciences & Peking Union Medical College Beijing China; ^3^ Peking University Fifth School of Clinical Medicine Beijing China; ^4^ Evidence‐Based Medicine Centre School of Basic Medical Sciences Lanzhou University Lanzhou China; ^5^ School of Population Medicine and Public Health Chinese Academy of Medical Sciences & Peking Union Medical College Beijing China; ^6^ State Key Laboratory of Respiratory Health and Multimorbidity Chinese Academy of Medical Sciences & Peking Union Medical College Beijing China; ^7^ Institute of Allergology Charité ‐ Universitätsmedizin Berlin Fraunhofer Institute for Translational Medicine and Pharmacology, Allergology and Immunology Berlin Germany

**Keywords:** asthma, life course, non‐pharmacological interventions, umbrella review

## Abstract

**Background:**

The impact of non‐pharmacological interventions (NPIs) on asthma prevention and management is insufficiently examined. We aim to comprehensively evaluate and synthesize existing evidence regarding the effectiveness of various NPIs throughout the life course.

**Methods:**

We conducted a systematic search and screening of reviews that examined the effectiveness of various NPIs on asthma prevention and control in the Cochrane Library, PubMed, Embase, and Ovid databases. Data extraction was performed by considering the type of NPIs and the life course stages of the target population. Recommendations were provided by considering the quality of review assessed using the AMSTAR2 tool and the consistency of findings across reviews.

**Results:**

We identified 145 reviews and mapped the evidence on the impact of 25 subtypes of NPIs on asthma prevention and control based on five stages of life course. Reviews indicated a shift of focus and various impacts of major NPIs on asthma prevention and control across life courses, while a few types of NPIs, such as physical exercise, appeared to be beneficial in children, adolescents and adults. Consistent and high‐level evidence was observed only for psychological intervention on asthma control and quality of life among adults and older adults. Potential benefit with high‐level evidence was reported on certain NPIs, such as vitamin D in reducing risk of developing asthma in offsprings in the prenatal stage, digital health interventions in improving asthma control from childhood to older adulthood, and breathing exercise in improving quality of life, asthma‐related symptoms and lung function in adulthood and older adulthood.

**Conclusion:**

This study emphasizes the significance of delivering NPIs to improve asthma prevention and management and highlights the heterogeneity regarding the impact of NPIs across life courses. High‐quality research is urgently needed to further strengthen the evidence base of NPIs and tailored interventions should be considered in guideline development.

## 1 INTRODUCTION

Asthma is a prevalent global health issue, imposing substantial healthcare costs related to hospitalization and medication. Despite a 24% decrease in disease burden since 1990, the prevalence of asthma remains high with about 262.4 million cases worldwide in 2019, resulting in an age‐standardized rate of 273.6 DALYs per 100,000 people.^1^ Asthma affects all age groups and presents enduring challenges for long‐term management, exerting a profound impact on individuals' quality of life.^2^

An increasing body of evidence has emerged about effective interventions to prevent and manage asthma, which cover both pharmacological and non‐pharmacological interventions (NPIs). NPIs refers to a wide range of measures aimed at mitigating exposures to risk factors or reinforcing the protective risk factors related to asthma by altering key aspects of changeable behaviours,^3^ holding the potential of enhancing asthma prevention and control. Although NPIs have been highlighted as key strategies for managing major chronic diseases,^4^, ^5^, ^6^, ^7^ the impact of NPIs is not fully understood and is insufficiently emphasized in the clinical guidelines and practice.

Asthma can manifest at any age, with distinct characteristics. The World Health Organization advocates for a life‐course approach to manage chronic conditions, placing a significant emphasis on fostering health and well‐being throughout all stages of life and recognizing the cumulative effects of managing risk factors on long‐term outcomes.^8^, ^9^ This approach offers a unique lens and intuitive framework for understanding effective strategies for asthma prevention and control. By employing this framework to systematically analyse existing evidence, we can discern both similarities and differences in characteristics and long‐term effectiveness across intervention strategies.

Despite the great potential of timely nonpharmacological interventions in asthma prevention and control, the evidence has not been systematically synthesized. To fill in such gaps, we conducted an umbrella review, also known as an overview of systematic review, aiming to assess and synthesize existing evidence on the effectiveness of various proposed NPIs for the prevention and management of asthma. By categorizing NPIs by different life stages, this review seeks to provide recommendations for both clinical practice and further research in the comprehensive management of asthma.

## 2 METHODS

### 2.1 Search strategy

This umbrella review employed a systematic approach to gather and evaluate information from multiple systematic reviews and meta‐analyses encompassing all types of NPIs for asthma.^10^, ^11^ The review protocol was registered with PROSPERO under the registration number CRD42023397295. Two reviewers, XZ and MW, conducted a comprehensive literature search of articles published between Jan 2010 and Dec 2022 (with the final search conducted on 9th February, 2023), by using the search algorithm covering key terms related to asthma and NPIs, as detailed in Table S1. Four major medical databases, including Cochrane Library, PubMed, Embase and Ovid, were searched, covering the key literature in the field. Two reviewers independently screened the titles and abstracts of all identified systematic reviews using the COVIDENCE platform. Subsequently, they assessed the full text of potentially eligible systematic reviews for inclusion. Any discrepancies encountered during the search and screening process were resolved through discussion with senior third reviewers (XT and EG).

### 2.2 Eligibility criteria

This umbrella review considered all reviews with a systematic search that focused on evaluating the effectiveness of NPIs for preventing or managing asthma at both the individual and population levels. The inclusion criteria were set based on the ‘PICOs’ design and have been summarized in Table 1. In brief, the included studies need to be a review study with a systematic search, targeted on individuals at different stages of life with or without asthma, contained any type of NPIs, and reported at least one of the outcome measures related to asthma occurrence or control. The exclusion criteria are (1) studies focusing on pharmacological interventions without the involvement of healthcare professionals or targeting the behaviours of healthcare professionals; (2) commentary papers or general literature review without systematic search; (3) studies that did not contain the key outcome measures of interests; (4) studies published in languages other than English; and (5) studies with no full‐text paper available.

**TABLE 1:** Criteria of included studies.

PICOS item		Included types
Population	Asthma or non‐asthma populations, and then assigned age groups according to the time when the interventions commenced	Prenatal period (before birth) Infancy (aged 0–2 years) Childhood (aged 3–12 years) Adolescence (aged 13–18 years) Adulthood (aged 19–65 years) Older adulthood (aged 65 years above)
Intervention	Supplements	N‐3 LC‐PUFA^†^ Folic acid Microecological regulator (prebiotic/probiotic/synbiotic) Vitamin D Antioxidant (vitamin C and E) Magnesium Infant formula
	Diet	Dietary pattern Dietary intake Breastfeeding
	Exercise	Physical exercise Breathing exercise
	Weight management	Non‐surgical weight management Bariatric surgery
	Self‐management support and health education	Digital health interventions Home‐based self‐management support School‐based self‐management support Community‐based self‐management support Clinical‐setting‐based self‐management support
	Psychological interventions	
	Environmental interventions	Home‐setting indoor environmental interventions Occupational setting environmental interventions
	Physiotherapy intervention	
	Others	
Comparison	Usual care/no intervention/placebo/alternative solution	
Outcome	Disease control	Asthma‐related symptoms Quality of life Asthma control level
	Risk of developing asthma	
	Aute exacerbation	Acute exacerbation Hospitalization Emergency department visit
	Lung function	FEV1/FEV1% PEF/PEFR FVC
	Patient's behaviours	Medication adherence
	Asthma‐related absenteeism	School absence/days off work
Study design	Reviews with systematic search	

Abbreviations: FEV1, forced expiratory volume in one second; FEV1%, forced expiratory volume in one second/forced vital capacity; FVC, forced vital capacity.; n‐3, LC‐PUFA; omega‐3, long‐chain polyunsaturated fatty acids; PEF, peak expiratory flow; PEFR, peak expiratory flow rate.

### 2.3 Data extraction and synthesis

Data extraction was independently performed by two investigators, XZ and MW. The basic information of the review article (including author, year of publication, review period, number of included studies, language restriction) and the key characteristics of interventions (such as targeted population, type of interventions, comparison, key findings on effectiveness) were extracted. The synthesis of major findings from the existing reviews was conducted and categorized by the type of intervention and population groups. Interventions were classified into seven categories, including supplements, diet, exercise, weight management, self‐management support and health education, psychological interventions, and environmental interventions (detailed in Table S2), while the health‐related outcomes were grouped into six categories to reflect different goals in asthma prevention and management (Table 1).

Studies were classified based on the type of NPIs, while studies that targeted individuals across multiple life‐course stages were analysed separately if results from subgroup analysis on different types of populations are available. The number of reviews targeted on each intervention type at different life‐course stages was counted. For the paired type of intervention and outcomes, we categorized the effect estimates into three levels to represent the effectiveness of interventions and the consistency of findings across reviews: ‘consistent evidence with benefit’ signifies unanimous positive effects across all studies, ‘inconsistent evidence with benefit’ indicates mixed findings of effectiveness with some evidence on the benefit, and ‘no significant benefit’ indicates consistently ineffective findings.

### 2.4 Quality assessment

The quality of the included studies was evaluated with the AMSTAR 2 (A MeaSurement Tool to Assess systematic Reviews) tool,^12^ which was independently performed by two reviewers, XZ and MW. We made an adjustment to AMSTAR2 to reflect the relatively low level of quality of identified reviews and re‐classified item 2,4,9, from three levels to two levels. The overall confidence in the results of each systematic review was then rated as high, moderate, low, or critically low.

### 2.5 Rules of recommendation

In order to obtain effective interventions with robust evidence, we collectively considered the quality of review assessed based on AMSTAR‐2 and the consistency of evidence based on intervention type and specific outcomes. Interventions with either consistent evidence or moderate‐‐high level quality evidence were considered in the recommendation list. These interventions were classified into three levels, including (1) moderate to high‐level quality with consistent benefit, (2) moderate to high quality with inconsistent but potential benefit and (3) low‐level quality with consistent benefit.

## 3 RESULTS

### 3.1 Study screening

**FIGURE 1:**
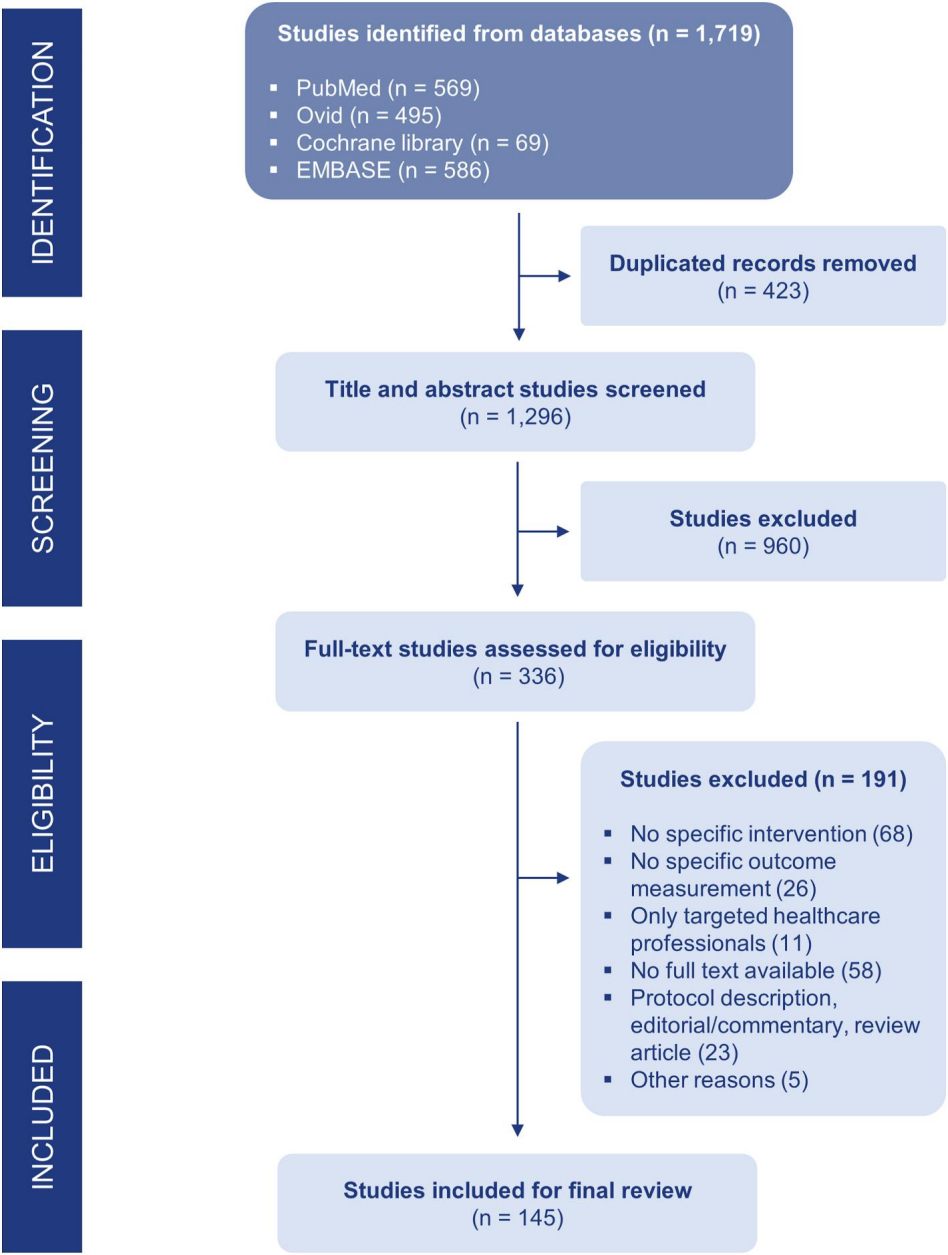
PRISMA flow diagram.

Figure 1 shows the process of study screening and inclusion. A total of 1719 reviews were initially identified from the search, with 569 reviews from PubMed, 495 from Ovid, 69 from the Cochrane library, and 586 from EMBASE. After removing duplicates and irrelevant articles based on titles and abstracts, 336 full‐text articles were further screened. About 191 reviews were further excluded due to various reasons such as not following the PICO criteria or not full‐text available, and then a total of 145 reviews and meta‐analysis (SRs/MAs) were included in this umbrella review.

### 3.2 Characteristics of the included SRs/MAs

The included SRs/MAs were conducted between 2011 and 2022, with a substantial proportion (*n* = 46, 31.7%) of studies published between 2020 and 2022. The number of original studies contained in the included SRs/MAs ranged from 2 to 117. Among these articles, 91 included randomized controlled trials only, 8 included non‐randomized trials, and 46 included both randomized and non‐randomized trials. Detailed characteristics of the 145 included studies are provided in Table S3.

**FIGURE 2:**
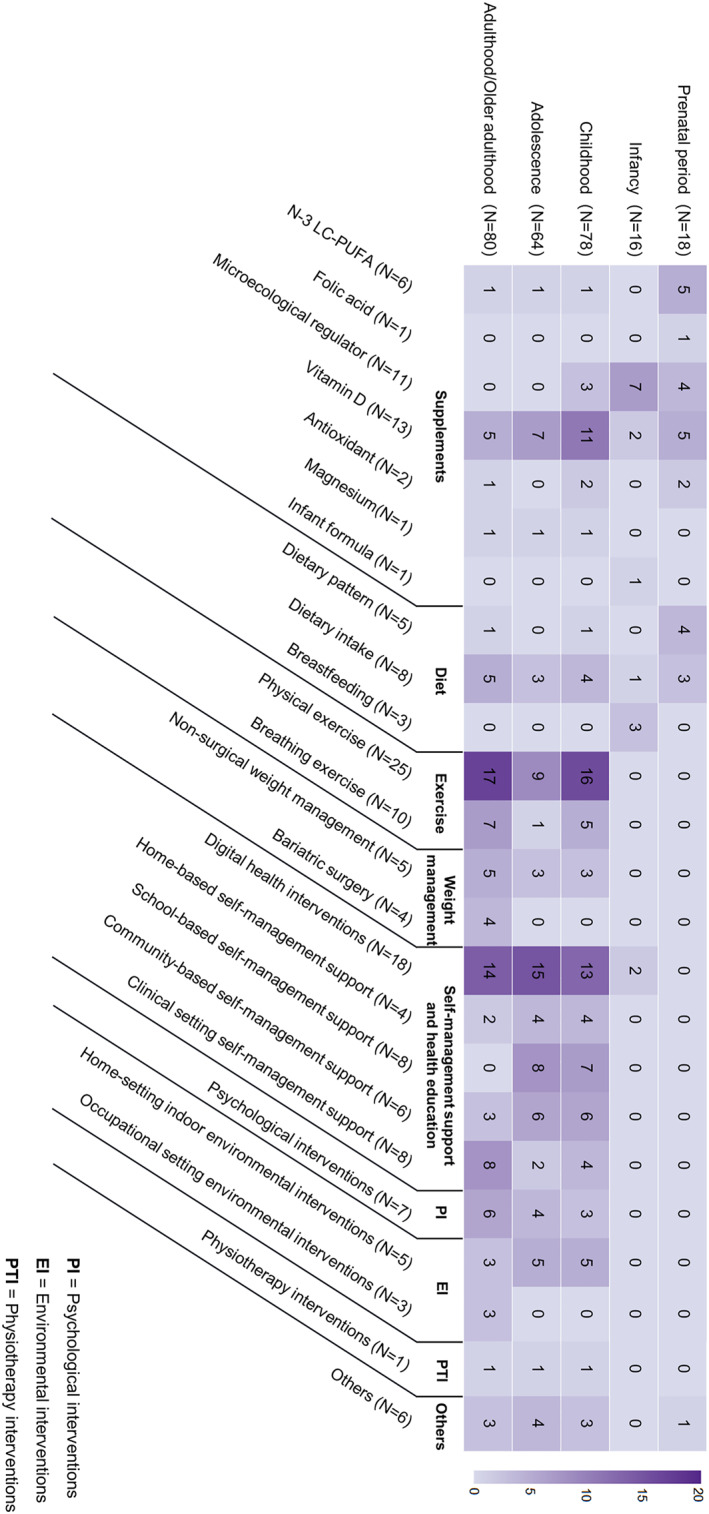
Number of included studies on non‐pharmacological interventions for asthma by stages of life course. The colour intensity corresponds to the number of included studies within each category, with deeper colours indicating higher article counts. Intervention categories are represented on the *X*‐axis, and life course stages are shown on the *Y*‐axis.

Figure 2 illustrates the focus of intervention types and the life courses among all included SRs/MAs. While many studies focused on multiple life course stages, more than half of the included reviews covered individuals in adulthood (80 reviews, significantly overlapped with older adulthood), childhood (78 reviews), and adolescence (64 reviews), with some reviews across multiple stages. The focus of the interventions examined in these reviews also varied greatly. Among all types of NPIs, 43 reviews focused on self‐management support and health education delivered from multiple means, 34 reviews examined the impact of supplements, 34 reviews targeted physical exercise or breathing exercise, followed by diet, weight management and other types of NPIs.

### 3.3 Effectiveness of interventions across life‐courses

**FIGURE 3:**
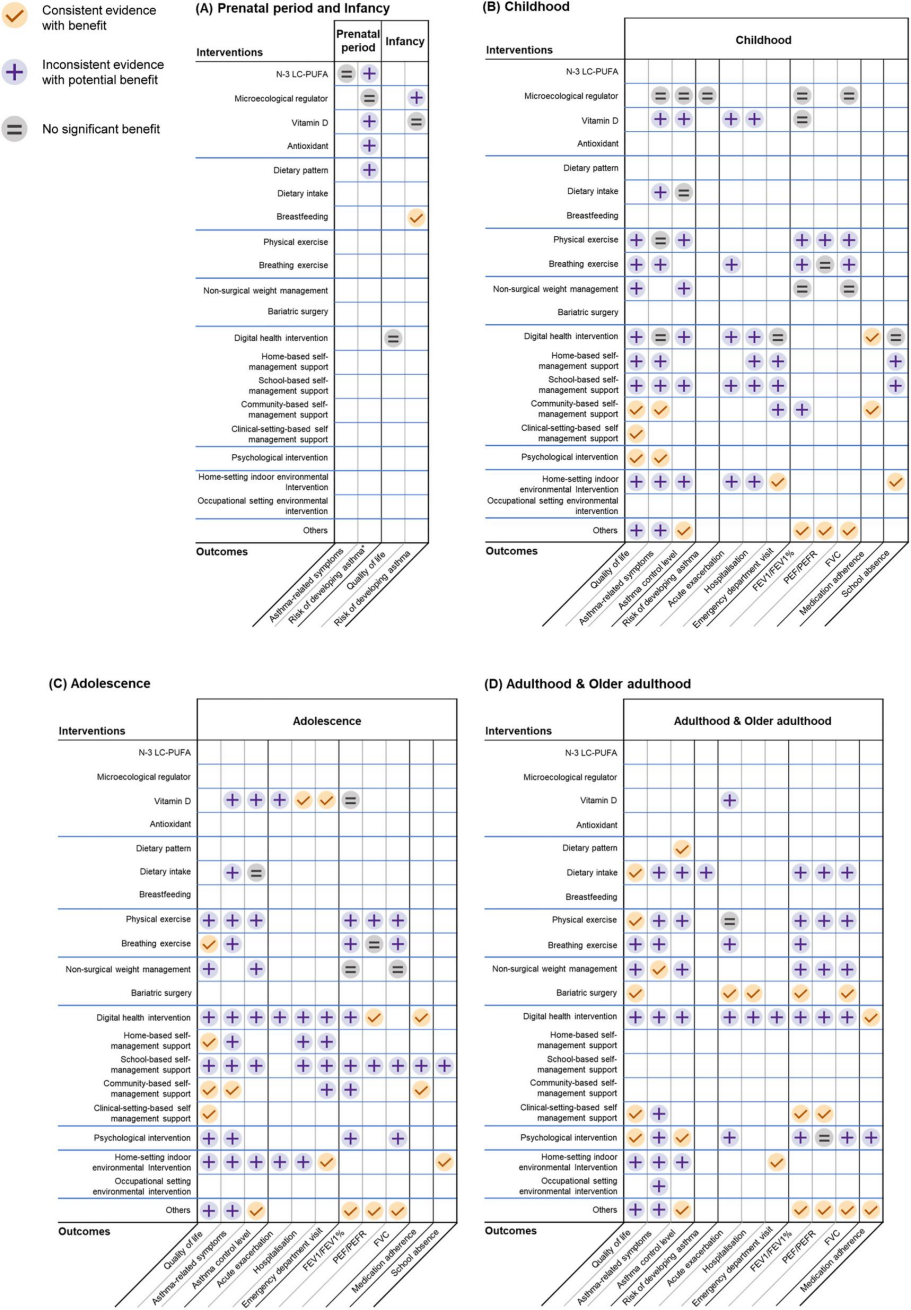
Effectiveness of key non‐pharmacological interventions (NPIs) across the life course. (A) Effectiveness of key NPIs in the prenatal period and infancy. (B) Effectiveness of key NPIs in childhood. (C) Effectiveness of key NPIs in adolescence. (D) Effectiveness of key NPIs in adulthood and older adulthood. *Risk of developing asthma, risk of developing asthma in the offspring. FEV1%, forced expiratory volume in one second/forced vital capacity; FEV1, forced expiratory volume in one second; FVC, forced vital capacity; n‐3 LC‐PUFA, omega‐3 long‐chain polyunsaturated fatty acids; PEF, peak expiratory flow; PEFR, peak expiratory flow rate.

NPIs displayed varying effectiveness across different life stages, as illustrated by different outcomes in Figure 3.

#### 3.3.1 Prenatal period

For the stage of prenatal period, supplements and diet emerged as the predominant types of NPIs examined in the reviews. Among the 18 SRs/MAs targeting pregnant women, 17 reviews focused on supplementation. Although several reviews reported the benefit of vitamin D, n‐3 LC‐PUFA and antioxidant supplementation in reducing the risk of developing asthma in offspring, the evidence remained inconsistent. Microecological regulators yield no significant benefit in reducing offspring's risk of developing asthma.

#### 3.3.2 Infancy

A total of 16 SRs/MAs included in the analysis examined the impact of NPIs among infants. Three reviews demonstrated the consistent benefit of exclusive breastfeeding in reducing the long‐term risk of developing asthma,^13^, ^14^, ^15^ and relatively strong evidence from a meta‐analysis supports exclusive breastfeeding of a duration ≥6 months.^15^ There was no significant benefit of vitamin D supplementation for reducing asthma risk during infancy.^16^, ^17^ Additionally, although seven reviews investigated the impact of microecological regulators, their effectiveness remains inconclusive.

#### 3.3.3 Childhood

About 78 SRs/MAs examined a wide range of NPIs among children, with physical exercise, digital health interventions and vitamin D consumption as the top three subtypes of interventions of interest. Eleven SRs/MAs assessed the effects of vitamin D, while findings were inconsistent with a few studies indicating its benefit on asthma‐related symptoms, asthma control level, acute exacerbation, and hospitalization. These inconsistent conclusions of asthma exacerbation may be attributed to the variations in serum 25(OH)D concentrations among asthmatic children. Two of the included reviews found that vitamin D supplementation significantly reduced the risk of asthma exacerbations only in the subgroup population whose serum 25(OH)D levels were low (<10 ng/mL for one review and <25 nmol/L for the other review).^18^, ^19^Besides, while microbial supplementation has been extensively studied in the paediatric context, our analyses revealed negligible effects on children.

A shift in the key intervention approaches has been observed from supplements and diet in the prenatal and infancy stages to exercise and weight management in the childhood stage. Both physical exercise and breathing interventions have shown inconsistent evidence with benefit in improving quality of life and lung function. Besides, some but inconsistent evidence has shown the positive impact of physical exercise on asthma control level, as well as breathing exercise on reducing asthma‐related symptoms and acute exacerbation of asthma. Non‐surgical weight reduction interventions exhibited inconsistent but potential effectiveness in improving asthma control level, yet demonstrated no significant benefit in lung function in children.

Notably, a large proportion of reviews focused on the impact of self‐management support and health education programs implemented in diverse settings that children inhabit, spanning homes, schools, and communities. These interventions in general have multiple components and demonstrated wide‐ranging benefits for asthma control. A total of 12 reviews emphasized the use of digital health solutions to support self‐management and health education, and showed consistent benefits in improving medication adherence and modest but inconsistent benefits in a variety of outcomes for asthma control.^20^, ^21^, ^22^, ^23^, ^24^, ^25^, ^26^, ^27^, ^28^, ^29^, ^30^, ^31^ Three reviews focused on psychological interventions and showed promise in paediatric asthma control, as consistent evidence from the included reviews demonstrated the effectiveness of psychological interventions, including motivational interviewing, problem‐solving skills, cognitive behaviour therapy, emotional disclosure and art therapy in improving quality of life and reducing symptoms.^25^, ^32^ Lastly, indoor environmental interventions in the home setting exhibit consistent benefits in reducing emergency department visits and school absenteeism for children with asthma.

#### 3.3.4 Adolescence

In the stage of adolescence, 64 SRs/MAs assessed the effects of NPIs and the findings were similar to those at the childhood stage. Despite slight differences in some outcomes, reviews showed similar and prolonged impact of some key NPIs, such as dietary intake, weight management, physical exercise, breathing exercise, community‐based interventions, and home‐setting indoor environmental interventions, among adolescents compared with childhood. This indicates that these key NPIs may continuously influence individuals with asthma and improve the long‐term outcomes in adjacent life course. Specifically, eight SRs/MAs showed the promise of school‐based self‐management support and health education programs with an assessment of series of asthma‐related outcomes with inconsistent evidence with benefits. One review examined the impact of breathing exercise and reported a positive impact of breathing exercise, including breathing exercise with yoga, Buteyko breathing techniques and physiotherapist‐led breathing training in improving the quality of life.^33^ However, as studies shared high heterogeneity in design, no conclusion has been reached on the type of breathing exercise, as well as the duration and intensity of exercise from the review.

#### 3.3.5 Adulthood and older adulthood

A total of 80 SRs/MAs assessed NPIs for adulthood and older adulthood stages, while only one review targeted older individuals aged 65 and above.^34^ Some NPIs, such as exercise, psychological interventions and digital health interventions, draw great attention in existing reviews with similar evidence of impacts from the earlier stage of life to adulthood. Six reviews focused on different types of psychological interventions and demonstrated the beneficial roles of psychological interventions on various outcomes, such as motivation interviews for improving medication adherence,^35^ meditation for reducing perceived stress and increasing quality of life,^36^ cognitive behavioural therapy for improving asthma control,^37^ etc. Besides, similar to the findings from the adolescent stage, a Cochrane review also reported the modest evidence on the improvement of quality of life among adults after 3‐months of breathing‐exercises, though high heterogeneity was identified across studies and dosage and types of breathing exercise sessions remained inconclusive.^38^ Furthermore, a meta‐analysis study compared the beneficial impact of different types of exercise and revealed that aerobic exercises were more beneficial than free‐choice or combined types of exercise for lung function and quality of life among asthma patients.^39^ Although evidence showed that high‐intensity interval training could reduce asthma symptoms during the chronic phase,^40^ no conclusion has been given in existing reviews on the duration and intensity of exercise.

Some key characteristics of interventions at adulthood are worthy of special attention. Reviews on supplementation interventions notably decreased in number, while a significant proportion of review articles examined and reported positive impacts of dietary patterns and diet intake on improving asthma control level, quality of life and lung function. Weight management interventions, particularly bariatric surgery, have emerged as significant strategies for adults with asthma, with four SRs/MAs reporting consistent benefits on quality of life, acute exacerbation of asthma, and lung function outcomes.^41^, ^42^, ^43^, ^44^, ^45^ Moreover, studies reported a consistent benefit of home‐setting indoor environmental interventions in reducing emergency department visits and modest but inconsistent evidence on reducing asthma‐related symptoms. For occupational asthma unique to adulthood, interventions addressing occupational setting environments prove inconsistent evidence with benefit in symptom reduction.

### 3.4 Methodological quality of included SRs/MAs

The AMSTAR 2 tool was used to evaluate the methodological quality of the included SRs/MAs. The majority of SRs/MAS (*n* = 109, 75.17%) were rated as critically low quality, with the remaining reviews being of low quality (*n* = 28, 19.31%), moderate quality (*n* = 3, 2.06%) and high quality (*n* = 6, 4.14%). The AMSTAR 2 assessment of included SRs/MAs is provided in Table S4.

### 3.5 Recommendation on effective interventions across the life course

**FIGURE 4:**
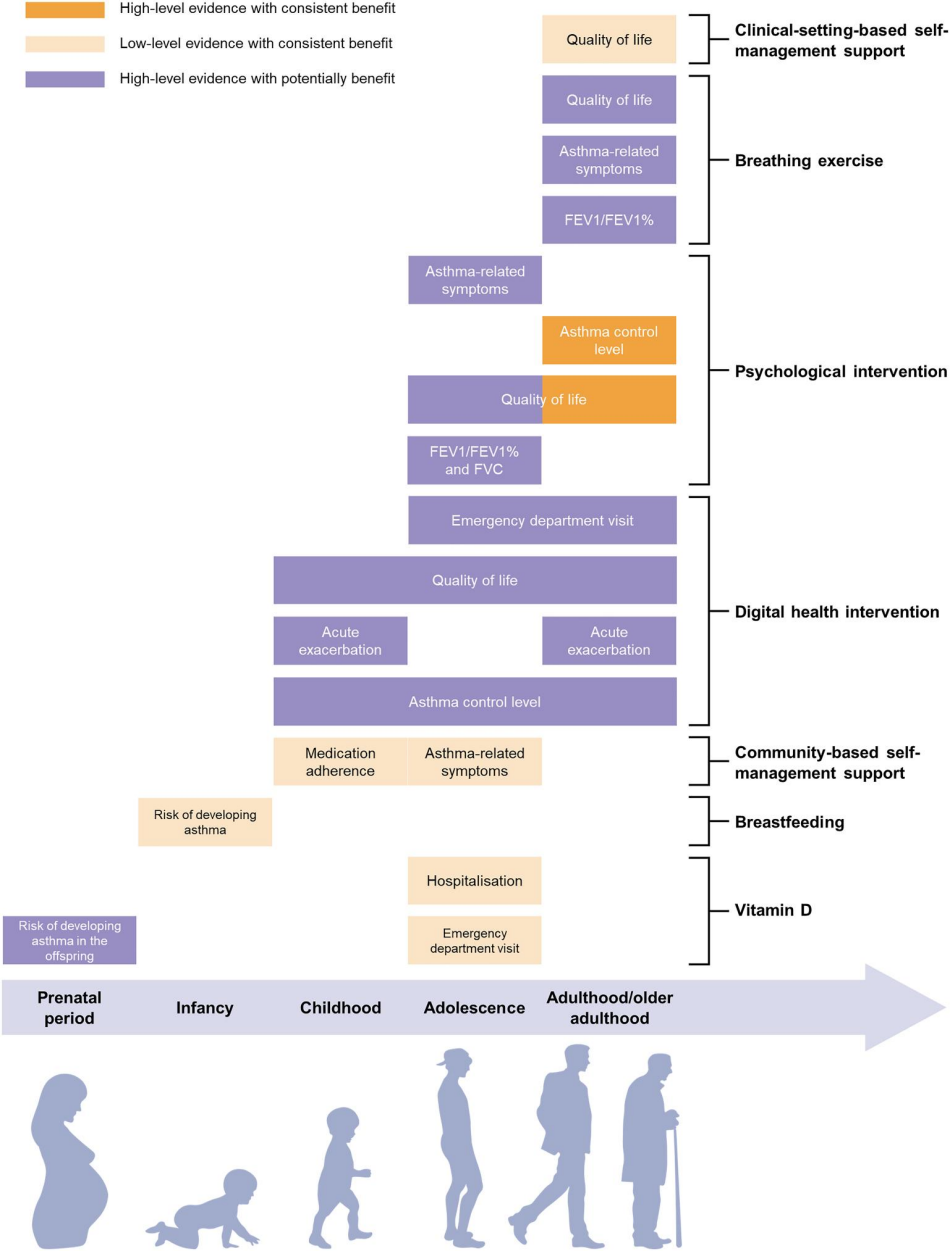
Recommendation of results across the life course. FEV1%, forced expiratory volume in one second/forced vital capacity; FEV1, forced expiratory volume in one second; FVC, forced vital capacity.

As shown in Figure 4, seven types of interventions with paired outcomes from 12 SRs/MAS were identified as recommended or suggested interventions for asthma prevention and control. Specifically, high‐level evidence with consistent benefit was observed for psychological intervention on asthma control and quality of life among adulthood and older adulthood,^46^ which was on the top of the recommendation list. Low‐level evidence with consistent benefit can be seen in several interventions, such as breastfeeding in reducing risk of developing asthma in infancy, community‐based self‐management support in improving medication adherence among children and reducing asthma‐related symptoms in adolescence, vitamin D in reducing hospitalisation and emergency department visit in adolescence, and clinical‐setting‐based self‐management support on the quality of life in adulthood/older adulthood. Furthermore, high‐quality reviews suggested the potential benefit of a number of pairs of interventions and outcomes, such as vitamin D in reducing the risk of developing asthma in the offspring in the prenatal stage, digital health interventions in improving asthma control and quality of life from childhood to older adulthood stages, and breathing exercise in improving quality of life, asthma‐related symptoms and lung function from adulthood and older adulthood stages. However, existing reviews did not provide consistent findings on their effectiveness; thus, we listed it at the bottom of the recommendation list to suggest its potential benefits and inadequate in consistency.

## 4 DISCUSSION

This umbrella review maps existing evidence about NPIs for asthma prevention and management following the life‐course approach. A total of 145 reviews were included in the analysis and the findings from the reviews were synthesized into seven major types and 25 sub‐types of NPIs, addressing various asthma related outcomes across the life course. These NPIs addressed a variety of behavioural, psychological and environmental factors related to asthma prevention and control across life course stages. However, we only identified 12 reviews with low to high methodology quality and reported the potential or consistent benefit of NPIs. Besides, many existing reviews remained inconclusive due to the limited amount of included literature and the relatively high heterogeneity in intervention design and outcome measures.

Our study suggests that NPIs should be a key component for the prevention and management of asthma throughout life courses. Different from a few existing reviews that focused solely on a specific life‐course stage,^47^ our review considers the whole life‐course and demonstrates a large variation in evidence in the focus of NPIs across life course stages. For instance, dietary and supplement interventions were major NPIs examined in prenatal and infant stages, while the research interests of NPIs were broader to cover exercise, weight management, self‐management support, psychological intervention and environmental interventions in childhood and adolescence stage, and then to environment factor control in home‐based or occupational settings. Such a shift in focus reflected the diverse interests, evidential variation and evolving needs across life course stages.

The importance of NPIs has been reinforced by the consistent effectiveness of NPIs on a variety of asthma‐related outcomes covering multiple life‐course stages. Among all recommended interventions, psychological interventions deserve special attention. In our review, psychological interventions covered a range of intervention strategies, such as motivational interviewing, meditation, and cognitive‐behavioural therapy, and have been shown to have consistent benefits in improving the quality of life in both childhood and adulthood stages and optimizing asthma control in adulthood.^25^, ^35^, ^36^, ^37^, ^46^, ^48^, ^49^ These findings highlight the great potential of integrating valued psychological intervention into self‐management programs and digital health solutions for asthma control to catalyse asthma control programme and improve the quality of life among patients.

In addition, our study found the beneficial roles of some NPIs beyond the single life‐course stage. For instance, controlling indoor environmental factors has been proven effective in reducing emergency department visits and school or working absences among children, adolescents, and adults.^50^, ^51^, ^52^, ^53^, ^54^ Similarly, existing reviews also indicate the beneficial roles of exercise in childhood, adolescence and adulthood. Although future studies should further understand the recommended dosage and types of exercise for each life‐course stage, exercise is one of the NPIs that may have prolonged impact. These findings encourage early implementation of interventions when feasible to prevent or slow down the development of asthma, as well as to enhance the accumulated impact of NPIs beyond life course stages for a prolonged benefit even across generations.

Notably, our study also revealed that certain NPIs may have distinct impacts on populations in different stages of life, which need special attention. For example, microbiome‐related interventions, though studied across prenatal, infancy, and childhood stages, have only shown inconsistent evidence of their benefit in the infancy stage.^55^, ^56^, ^57^, ^58^, ^59^, ^60^, ^61^, ^62^ Weight management is significant in adulthood with relatively strong evidence indicating its effectiveness in enhancing quality of life, reducing acute exacerbation and improving lung functions,^42^, ^43^, ^44^, ^63^, ^64^, ^65^, ^66^, ^67^ while its impact has been reported as non‐significant for improving lung function among children and adolescents.^65^, ^66^, ^67^ This heterogeneity in findings across life courses highlights the special needs to implement tailored NPIs based on their life courses and individual goals, and suggests that recommendations in guidelines and practice should be provided by considering the diverse impact of interventions on various health outcomes and target population.

This umbrella review also provides important insights and implications for clinical practice, guideline development and research activities. As exemplified by the 2023 GINA Global Strategy for Asthma Management and Prevention, our review underscores the growing body of evidence supporting NPIs, to supplement pharmacological treatment and optimize asthma prevention and control and to enhance routine clinical practice. On the other hand, our review also indicates some major gaps in evidence and suggests that future research should investigate the reasons and underlying mechanisms of the diverse impact of NPIs across populations and the accumulated long‐term effectiveness of NPIs across multiple life‐course phases. As many of the reviews failed to reach a conclusion about standardized recommended NPIs with the greatest benefits, high‐quality research is needed to understand the impact of different intervention dosages and delivery approaches on both short‐term and long‐term outcomes for asthma control.

To the best of our knowledge, this umbrella review is the first to comprehensively evaluate the effectiveness of NPIs for asthma across the entire life course, offering a novel and valuable contribution to the field. This wide‐ranging approach allows for a more extensive comparison of evidence, facilitating a better understanding of strategies for asthma prevention and management. Still, some limitations of this study should be acknowledged. Firstly, due to the set of our search strategies, review articles published in non‐English language, before 2010, or not included in the major key database were excluded from the review. Secondly, as a common limitation of the umbrella review, the evidence was generated by synthesizing findings from existing reviews; thus, details of the original trial studies were potentially overlooked in this umbrella review and there might be overlaps of included studies across different review articles. In addition, although we synthesized similar interventions in groups, we have to acknowledge the heterogeneity in intervention design, delivery approach, context examined and outcomes assessed in both original studies and existing reviews. The current classification of interventions emphasizes the similarity of interventions based on their intervention purposes and targets, while interventions share high heterogeneity in dosage, delivery approach, intensity and duration. Such diversity in nature adds difficulties in conducting quantitative synthesis and direct comparisons in existing reviews, as well as in this umbrella review. Thus, high‐quality research is needed to compare different means of intervention delivery of major NPIs and provide evidence‐based recommendations about the beneficial dosage of NPIs.

## 5 CONCLUSIONS

This study reinforced the importance of NPIs, especially psychological interventions, for a life‐course strategy of asthma prevention and control. The diverse benefit of some NPIs across life‐course suggests the need for tailored intervention strategies in clinical practice. More high‐quality studies are needed to understand the different impacts of NPIs across populations and to identify strategies to provide personalized, effective and cost‐effective interventions to optimize asthma prevention and control.

## CONFLICT OF INTEREST STATEMENT

The authors have none to declare.

## Supporting information

Table S1

Table S2

Table S3

Table S4

## Data Availability

The data that support the findings of this study are available in the supplementary material of this article.
