# Supplementary material for: Non‐pharmacological interventions for asthma prevention and management across the life course: Umbrella review
Source: Clin Transl Allergy. 2024 Feb 29;14(3):e12344. doi: 10.1002/clt2.12344 (PMC10904350; doi:10.1002/clt2.12344)
Supplement: Supplementary file 1 — Table S1 [file CLT2-14-e12344-s004.docx]

**Table S1 Search terms and number of papers identified.**

| **Data base** | **Search terms** | **Records identified (N)** |
| --- | --- | --- |
| PubMed | (("Non-pharmacological"[Title/Abstract] OR "non drug*"[Title/Abstract] OR ("Life Style"[Title/Abstract] OR "Behavior Therapy"[Title/Abstract] OR "Exercise"[Title/Abstract] OR "Smoking Cessation"[Title/Abstract] OR "Diet"[Title/Abstract] OR "Dietary Supplements"[Title/Abstract] OR "Body Weight"[Title/Abstract] OR "Breast Feeding"[Title/Abstract] OR ("Life Style"[MeSH Terms] OR "Behavior Therapy"[MeSH Terms] OR "Exercise"[MeSH Terms] OR "Smoking Cessation"[MeSH Terms] OR "Diet"[MeSH Terms] OR "Dietary Supplements"[MeSH Terms] OR "Body Weight"[MeSH Terms] OR "Breast Feeding"[MeSH Terms])) OR ("Self-Management"[Title/Abstract] OR "Self Care"[Title/Abstract] OR "Health Education"[Title/Abstract] OR "Social Support"[Title/Abstract] OR ("Self-Management"[MeSH Terms] OR "Self Care"[MeSH Terms] OR "Health Education"[MeSH Terms] OR "Social Support"[MeSH Terms])) OR ("allergen avoidance"[Title/Abstract] OR "Environmental Exposure"[Title/Abstract] OR "Environmental Exposure"[MeSH Terms]) OR ("internet based*"[Title/Abstract] OR "web based*"[Title/Abstract] OR "Telerehabilitation"[Title/Abstract] OR "Mobile application"[Title/Abstract] OR ("Telerehabilitation"[MeSH Terms] OR "Mobile Applications"[MeSH Terms]))) AND ("asthma*"[Title] OR "Asthma"[MeSH Terms]) AND ("Systematic Review"[Publication Type] OR "Meta-Analysis"[Publication Type] OR ("systematic"[Title/Abstract] AND "review*"[Title/Abstract]))) AND (2010:2022[pdat]) | 569 |
| Cochrane library | ((Non-pharmacological):ti,ab,kw OR ("non drug*"):ti,ab,kw) OR (("Life Style"):ti,ab,kw OR ("Behavior Therapy"):ti,ab,kw OR (Exercise):ti,ab,kw OR ("Smoking Cessation"):ti,ab,kw OR (Diet):ti,ab,kw) OR (("Dietary Supplements"):ti,ab,kw OR ("Body Weight"):ti,ab,kw OR ("Breast Feeding"):ti,ab,kw) OR (("Self-Management"):ti,ab,kw OR ("Self Care"):ti,ab,kw OR ("Health Education"):ti,ab,kw OR ("Social Support"):ti,ab,kw) OR (("allergen avoidance"):ti,ab,kw OR ("Environmental Exposure"):ti,ab,kw) OR (("internet based*"):ti,ab,kw OR ("web based*"):ti,ab,kw OR (Telerehabilitation):ti,ab,kw OR ("Mobile application"):ti,ab,kw) OR (MeSH descriptor: [Life Style] explode all trees) OR (MeSH descriptor: [Behavior Therapy] explode all trees) OR (MeSH descriptor: [Exercise] explode all trees) OR (MeSH descriptor: [Smoking Cessation] explode all trees) OR (MeSH descriptor: [Dietary Supplements] explode all trees) OR (MeSH descriptor: [Body Weight] explode all trees) OR (MeSH descriptor: [Breast Feeding] explode all trees) OR (MeSH descriptor: [Self-Management] explode all trees) OR (MeSH descriptor: [Self Care] explode all trees) OR (MeSH descriptor: [Health Educators] in all MeSH products) OR (MeSH descriptor: [Social Support] explode all trees) OR (MeSH descriptor: [Environmental Exposure] explode all trees) OR (MeSH descriptor: [Telerehabilitation] explode all trees) OR (MeSH descriptor: [Mobile Applications] explode all trees) AND ((MeSH descriptor: [Asthma] explode all trees) OR ((asthma*):ti,ab,kw))  Limit Publication Date from 01/01/2010 – 31/12/2022 | 69 |
| Embase | (('non pharmacological':ab,ti OR 'non drug*':ab,ti) OR ('life style':ab,ti OR 'behavior therapy':ab,ti OR exercise:ab,ti OR 'smoking cessation':ab,ti OR diet:ab,ti OR 'dietary supplements':ab,ti OR 'body weight':ab,ti OR 'breast feeding':ab,ti) OR ('self-management':ab,ti OR 'self care':ab,ti OR 'health education':ab,ti OR 'social support':ab,ti) OR ('allergen avoidance':ab,ti OR 'environmental exposure':ab,ti) OR ('telerehabilitation'/exp OR 'mobile application'/exp)) AND (asthma*:ab,ti OR 'asthma'/exp) AND ((systematic:ab,ti AND review*:ab,ti) OR ('meta analysis'/exp OR 'systematic review'/exp)) NOT (((('non pharmacological':ab,ti OR 'non drug*':ab,ti) OR ('life style':ab,ti OR 'behavior therapy':ab,ti OR exercise:ab,ti OR 'smoking cessation':ab,ti OR diet:ab,ti OR 'dietary supplements':ab,ti OR 'body weight':ab,ti OR 'breast feeding':ab,ti) OR ('self-management':ab,ti OR 'self care':ab,ti OR 'health education':ab,ti OR 'social support':ab,ti) OR ('allergen avoidance':ab,ti OR 'environmental exposure':ab,ti) OR ('telerehabilitation'/exp OR 'mobile application'/exp)) AND (asthma*:ab,ti OR 'asthma'/exp) AND ((systematic:ab,ti AND review*:ab,ti) OR ('meta analysis'/exp OR 'systematic review'/exp))) AND [medline]/lim) AND [2010-2022]/py | 586 |
| Ovid | ((Non-pharmacological.ab. or Non-pharmacological.ti. or non-drug*.ab. or non-drug*.ti. or life style.ab. or life style.ti. or behavior therapy.ab. or behavior therapy.ti. or Exercise.ab. or Exercise.ti. or smoking cessation.ab. or smoking cessation.ti. or diet.ab. or diet.ti. or dietary supplement.ab. or dietary supplement.ti. or body weight.ab. or body weight.ti. or breast feeding.ab. or breast feeding.ti. or self-management.ab. or self-management.af. or health education.ti. or social support.ab. or social support.ti. or allergen avoidance.ab. or allergen avoidance.ti. or environmental exposure.ab. or environmental exposure.ti. or Internet-based*.ab. or Internet-based*.ti. or web-based*.ab. or web-based*.ti. or Telerehabilitation.ab. or Telerehabilitation.ti. or Mobile application.ab. or Mobile application.ti.) OR (life style or behavior therapy or Exercise or smoking cessation or diet or dietary supplement or body weight or breast feeding or self-management or health education or social support or environmental exposure or Telerehabilitation or Mobile application).sh.) AND (Asthma.sh. or Asthma.ab. or Asthma.ti.) AND (((systematic.ti. or systematic.ab.) AND (review.ti. or review.ab.)) OR (Systematic Review or Meta-Analysis).pt.) AND 2010:2022.(sa_year). | 495 |
