# Supplementary material for: Non‐pharmacological interventions for asthma prevention and management across the life course: Umbrella review
Source: Clin Transl Allergy. 2024 Feb 29;14(3):e12344. doi: 10.1002/clt2.12344 (PMC10904350; doi:10.1002/clt2.12344)
Supplement: Supplementary file 2 — Table S2 [file CLT2-14-e12344-s001.docx]

**Table S2.** **Descriptions of included nonpharmacological interventions for asthma**

| **Type of intervention** | | **Description** |
| --- | --- | --- |
| Supplements | N-3 LC-PUFA | Supplementation with omega-3 long-chain polyunsaturated fatty acids (through oily fish or fish oil) |
|  | Folic acid | Supplementation with folic acid |
|  | Microecological regulator (prebiotic/probiotic/synbiotic) | Administration of probiotics, prebiotics, or synbiotics |
|  | Vitamin D | Supplementation with vitamin D_3_ or vitamin D_2_ |
|  | Antioxidant (vitamin C and E) | Supplementation with vitamin C and vitamin E antioxidants |
|  | Magnesium | The use of oral magnesium as an adjunct to asthma inhalers |
|  | Infant formula | Hydrolysed formula (including extensively or partially hydrolysed formula) prescribed for infants |
| Diet | Dietary pattern | Consumption of a combination of foods from various groups (e.g., Mediterranean diet) |
|  | Dietary intake | Nutrition intervention or food intake |
|  | Breastfeeding | Ever or prolonged infant breastfeeding |
| Exercise | Physical exercise | Regular physical activity or a physical training programme, such as walking, running, cycling, swimming or other aerobic and low-intensity exercise (i.e. yoga), weight-bearing exercise, and exercise-based pulmonary rehabilitation |
|  | Breathing exercise | An approach for correcting dysfunctional breathing, can be performed as the Papworth method, Buteyko breathing technique, yoga or any other similar intervention that manipulates the breathing pattern |
| Weight management | Non-surgical weight management | Dietary restriction, exercise or physical activity for weight loss in overweight or obese patients with asthma |
|  | Bariatric surgery | A set of surgical procedures designed to affect metabolism and achieve significant weight reduction in individuals affected by severe obesity |
| Self-management support and health education | Digital health intervention | The use of digital technologies to deliver healthcare services, monitor health conditions, provide health information, or support individuals in managing their illness and well-being |
|  | Home-based self-management support | Self-management educational interventions for asthma self-management delivered in home environment to children, caregivers or both |
|  | School-based self-management support | Self-management educational interventions for asthma self-management delivered in school environment |
|  | Community-based self-management support | Socially and behaviourally focused programs occurring outside the clinical setting |
|  | Clinical-setting-based self-management support | Educational interventions carried out by any health care professionals in clinical setting |
| Psychological interventions | | Interventions focused on the management of stress, anxiety and/or depression, such as motivational interviewing, meditation, cognitive-behavioural therapy, problem-solving skills, emotional disclosure and art therapy |
| Environmental interventions | Home-setting indoor environmental interventions | Interventions to reduce exposure to indoor environmental allergens at home |
|  | Occupational setting environmental interventions | Work place interventions for sensitizer-induced occupational asthma, including removal from exposure and reduction of exposure |
| Physiotherapy interventions | | Physiotherapy uses massages and physical treatments to relieve pain, enhance movement, and strengthen muscles |
| Others | | Multiple interventions aimed at managing asthma by changing patients’ behaviours |
