# Supplementary material for: Non‐pharmacological interventions for asthma prevention and management across the life course: Umbrella review
Source: Clin Transl Allergy. 2024 Feb 29;14(3):e12344. doi: 10.1002/clt2.12344 (PMC10904350; doi:10.1002/clt2.12344)
Supplement: Supplementary file 3 — Table S3 [file CLT2-14-e12344-s002.docx]

**Table S3 Characteristics of the included systematic reviews and meta-analyses.**

| **Author, Year** | **Prenatal** | **Infancy** | **Childhood** | **Adolescence** | **Adulthood & Older people** | **Searching period** | **No of studies in each SR/MA** | **Type of included studies** | **Intervention type** | **Intervention subtype** | **Comparison** | **Outcomes** |
| --- | --- | --- | --- | --- | --- | --- | --- | --- | --- | --- | --- | --- |
| Gungor, 2019 |  | √ |  |  |  | 1980–2015 | 73 | RCT; nonrandomized controlled trials; prospective cohort study; retrospective cohort study; case-control study | Diet | Breastfeeding | No breastfeeding; shorter duration of breastfeeding | Incidence of asthma in childhood/adolescence |
| Dogaru, 2014 |  | √ |  |  |  | –2012 | 117 | Cohort; cross-sectional; case-control | Diet | Breastfeeding | Less breastfeeding | Incidence of asthma/wheeze in children |
| Xue, 2021 |  | √ |  |  |  | –2020 | 42 | Cohort study | Diet | Breastfeeding | Shorter duration of breastfeeding; less breastfeeding; no breastfeeding | Risk of asthma; incidence of asthma |
| Hosseini, 2017 |  |  | √ | √ | √ | –2016 | 17 | Cohort; case-control; cross-sectional; experimental trials | Diet | Dietary intake | NA | Asthma-related symptoms |
|  |  |  |  |  |  |  | 41 |  |  |  |  |  |
| Nurmatov, 2011 | √ | √ | √ |  |  | 1988–2009 | 62 | Cohort, case-control, cross-sectional | Diet; Supplements | Vitamin D; antioxidant; dietary pattern; dietary intake | NA | Incidence of asthma |
| Pogson, 2011 |  |  |  |  | √ | –2010 | 9 | RCT | Diet | Dietary intake | Placebo | Asthma control; lung function |
| Forte, 2018 |  |  |  |  | √ | 1948–2014 | 21 | RCT | Diet; supplements; weight management | Non-surgical weight management; dietary pattern; antioxidant; n-3 LC-PUFA | NA | Asthma control; quality of life; lung function |
| Crocker, 2011 |  |  | √ | √ | √ | 1966–2008 | 23 | RCT | Environmental intervention | Home-setting indoor environmental interventions | NA | Asthma-related symptoms; school absence; emergency visit |
| Welker, 2018 |  |  | √ | √ |  | 2000–2017 | 11 | RCT; NRCT | Environmental intervention | Home-setting indoor environmental interventions | NA | Asthma-related symptoms; hospital visits |
| Leas, 2018 |  |  | √ | √ | √ | –2017 | 67 | RCT; NRCT | Environmental intervention | Home-setting indoor environmental interventions | Placebo, other interventions | Asthma symptoms; asthma control; quality of life; asthma exacerbation, hospitalization |
| Henneberger, 2019 |  |  |  |  | √ | 2010–2019 | 26 | RCT | Environmental intervention | Occupational setting environmental interventions | No intervention | Asthma-related symptoms; FEV1 % |
| Henneberger, 2021 |  |  |  |  | √ | –2019 | 18 | NRCT | Environmental intervention | Occupational setting environmental interventions | No intervention | Asthma-related symptoms; FEV1 %; Risk of unemployment |
| Dokbua, 2018 |  |  | √ | √ |  | 2017–2017 | 12 | RCT; NRCT | Self-management support and health education | Self-management support | NA | Asthma-related symptoms; quality of life; medication adherence |
| Ahmed, 2018 |  |  | √ | √ | √ | –2015 | 17 | RCT | Self-management support and health education | Self-management support | Usual care | Clinical outcomes, process outcomes, behavioral outcomes |
| Uchima, 2019 |  |  | √ | √ |  | 2017–2018 | 7 | RCT | Self-management support and health education | Education-based interventions delivered by health professionals | Usual care; no intervention | Quality of life; asthma-related symptoms; emergency visit; hospitalization |
| Chan, 2021 |  |  | √ | √ |  | 2000–2019 | 21 | Pre-post intervention study; RCT; cohort study | Self-management support and health education; Environmental intervention | Self-management; home-setting indoor environmental interventions | No intervention | Emergency visit; hospitalization; asthma control; FEV1 |
| Hodkinson, 2020 |  |  | √ | √ | √ | 2000–2019 | 105 | RCT | Self-management support and health education | Multidisciplinary case management  Regularly supported self-management  Regularly supported self-management  Multidisciplinary case management  Regularly supported self-management  Regularly supported self-management | Usual care | Quality of life |
| Pinnock, 2015 |  |  | √ |  | √ | 1980–2012 | 18 | Randomized trials; quasi-experimental study; historical controls; retrospective comparators | Self-management support and health education | Self-management | Usual care | Clinical outcomes |
| Press, 2012 |  |  |  |  | √ | 1950–2010 | 24 | No limitation | Self-management support and health education | Educational program | NA | Asthma-related symptoms |
| Peytremann-Bridevaux, 2015 |  |  |  |  | √ | 2000–2012 | 20 | Cluster-RCT; NRCT; and controlled before-after study | Self-management support and health education | Self-management support and health education to patients | Usual care | Quality of life; asthma severity; lung function; hospitalization; emergency visit |
| Welsh, 2011 |  |  | √ | √ |  | –2011 | 12 | RCT | Self-management support and health education | Home-based self-management support | NA | Emergency visits; quality of life |
| Mosnaim, 2017 |  |  | √ | √ |  | 2015–2016 | 6 | RCT | Self-management support and health education | Home-based self-management support | Usual care; NA | Asthma-related symptoms; asthma control; emergency visit; hospitalization; school absence; lung function |
| Schuers, 2019 |  |  | √ | √ | √ | –2017 | 22 | Clinical trial reports, literature reviews and meta-analyses | Self-management support and health education; environmental intervention; diet; exercise | Patient education programmes; multifaceted interventions; reduce exposure to allergens and indoor pollutants; air filtration systems; reduce exposure to dust mites; dietary interventions; promoting physical activity | NA | Asthma control |
| Fidler, 2021 |  | √ | √ | √ |  | 1998–2019 | 33 | RCT; NRCT | Self-management support and health education | Adherence-enhancing interventions | Usual care; asthma education; attention control | Medication adherence |
| Normansell, 2017 |  |  | √ | √ | √ | –2016 | 39 | RCT | Self-management support and health education | Education about adherence; using electronic monitoring or reminders to take the inhaler; making the drug easier to take; giving the inhaler during school hours | Usual care | Medication adherence |
| Gesinde, 2018 |  |  | √ | √ | √ | 2001–2016 | 11 | Intervention study | Self-management support and health education | Motivational interviewing | NA | Medication adherence |
| Garagorri-Gutierrez, 2022 |  |  | √ | √ | √ | 2014–2019 | 12 | Intervention study | Self-management support and health education exercise | Physiotherapy treatment | NA | Asthma control |
| Lee, 2021 |  |  | √ | √ | √ | –2016 | 33 | RCT; quasi-RCT and non-RCT | Self-management support and health education | Education; self-management plans; yoga; breathing exercises; organizational interventions; diet therapy; combined interventions | Usual care | Asthma symptoms; quality of life; FEV, FVC, FEV1, FVC ratio, PEF, and PEFR |
| Liao, 2019 |  |  | √ | √ | √ | –2019 | 9 | RCT; before-after study | Self-management support and health education | Goal setting method in intervention | Usual care | Symptom control; quality of life |
| Mosnaim, 2016 |  |  |  | √ |  | 2000–2014 | 21 | Intervention study | Self-management support and health education | Complex interventions with multiple behavioral interventions | NA | Quality of life, asthma-related symptoms; medication adherence; asthma control; lung function; exacerbation |
| Denford, 2014 |  |  |  |  | √ | 1998–2012 | 38 | RCT | Self-management support and health education | Self-care interventions | NA | Asthma-related symptoms; medication adherence |
| Maricoto, 2019 |  |  |  |  | √ | –2017 | 8 | RCT; quasi experimental | Self-management support and health education | Inhaler technique education | Placebo | Exacerbation |
| Harris, 2019 |  |  | √ | √ |  | –2017 | 33 | Experimental or quasi-experimental design; RCT | Self-management support and health education | School-based self-management support | No intervention | Hospitalization; emergency visit; school absence; quality of life |
| Kneale, 2019 |  |  | √ | √ |  | 1995–2018 | 33 | RCT | Self-management support and health education | School-based self-management support | NA | Emergency visit; hospitalization; school absence |
| Isik, 2019 |  |  | √ | √ |  | 2013–2018 | 8 | RCT and quasi-experimental | Self-management support and health education | School-based self-management support | NA | Emergency visit; hospitalization; school absence/days off work |
| Ramdzan, 2021 |  |  | √ |  |  | 1992–2019 | 23 | RCT; NRCT; uncontrolled pre-and post- study | Self-management support and health education | School-based self-management support | Usual care | School absence; asthma control |
| Walter, 2016 |  |  | √ | √ |  | –2015 | 6 | RCT | Self-management support and health education | School-based self-management support | Usual care | Quality of life; exacerbation; school absence/days off work; emergency visit |
| Ahmad, 2011 |  |  | √ | √ |  | 1998–2009 | 9 | RCT; pre-posttest; clustered RCT; quasi-experimental | Self-management support and health education | School-based self-management support | NA | School absence; emergency visit; hospitalization |
| Carvalho Coelho, 2016 |  |  | √ | √ |  | 2005–2014 | 17 | RCT; NRCT | Self-management support and health education | Educational asthma interventions | NA | Asthma-related symptoms; hospitalization; emergency visit; school absence; quality of life |
| Kew, 2017a^1^ |  |  | √ |  | √ | –2016 | 4 | RCT | Self-management support and health education | Shared decision-making for adults and children with asthma | Usual care | Quality of life; medication adherence; asthma control; emergency visit |
| Pinnock, 2017 |  |  | √ | √ | √ | 1993–2012 | 27 | RCT | Self-management support and health education | Self-management support interventions | Usual care | Asthma control; quality of life |
| Carson, 2013 |  |  | √ | √ | √ | –2013 | 21 | RCT | Exercise | Physical exercise | NA | Asthma-related symptoms; FEV1; FVC; PEFR; quality of life |
| McCallum, 2017 |  |  | √ | √ | √ | 1950–2016 | 7 | RCT | Self-management support and health education | Culture-specific programs for children and adults from minority groups with asthma | Usual care | Exacerbation; hospitalization; asthma control; quality of life |
| Hu, 2022 |  |  | √ | √ |  | –2021 | 20 | Original study | Environmental intervention | Home-setting indoor environmental interventions | NA | Asthma-related symptoms |
| Song, 2021 |  |  |  |  | √ | 2003–2020 | 22 | RCT | Self-management support and health education | Blended intervention：eHealth/face-to-face intervention | eHealth intervention  with or without usual care (UC) and face-to-face intervention  with or without UC or only UC | Lung function; quality of life; asthma control |
| Villa-Roel, 2016 |  |  |  |  | √ | 2001–2013 | 5 | RCT | Self-management support and health education | Educational interventions to increase primary care follow-up for adults seen in the emergency department | Usual care | Primary care follow-up; Reductions of relapses; Reductions of admissions |
| Gatheral, 2017 |  |  |  |  | √ | –2016 | 15 | RCT | Self-management support and health education | Personalized asthma action plans alone | Usual care | Emergency visit; hospitalization; asthma-related symptoms; quality of life; exacerbation; FEV1; school absence/days off work |
| Kew, 2017b^2^ |  |  |  | √ |  | –2016 | 5 | Parallel RCT | Self-management support and health education | School-based self-management support | Usual care; no intervention | Quality of life; exacerbation; asthma control; medication adherence; emergency visit |
| deGroene, 2011 |  |  |  |  | √ | 1996–2009 | 21 | RCT | Environmental intervention | Occupational setting environmental interventions | No intervention | Asthma-related symptoms; FEV1 % |
| Zhong, 2017 |  |  |  | √ |  | –2016 | 4 | RCT | Self-management support and health education | School-based self-management support | Adult, professional-led self-management support; usual care | Quality of life; lung function; asthma-related symptoms |
| Khalooeifard, 2021 |  |  |  |  | √ | 2008–2019 | 7 | RCT; NRCT | Weight management | Bariatric surgery | NA | FVC; FEV1; quality of life |
| Upala, 2019 |  |  |  |  | √ | –2018 | 6 | Randomized controlled trials or observational study | Weight management | Bariatric surgery | Non-surgical weight management; no control group | FEV1; FVC |
| Hossain, 2021 |  |  |  |  | √ | 1993–2019 | 39 | RCT; Case series and cohort study | Weight management | Bariatric Surgery | Usual care | Medication adherence; Hospitalization; Emergency visit; Lung function |
| Juel, 2012 |  |  |  |  | √ | –2012 | Not mentioned | RCT; NRCT | Weight management | Non-surgical weight management | NA | Asthma related symptoms; Quality of life; Hospitalization; Emergency visit; Lung function; Medication adherence |
| Okoniewski, 2019 |  |  | √ | √ | √ | 2000–2018 | 10 | 4 RCT for children | Weight management | Non-surgical weight management | NA | Asthma related symptoms; Quality of life; Lung function |
|  |  |  |  |  |  |  |  | 6 RCT involving adults |  |  |  |  |
| Lv, 2015 |  |  | √ | √ | √ | 1950–2014 | 7 | 3 RCT in children and adolescents | Weight management | Non-surgical weight management | Usual care | Disease control |
|  |  |  |  |  |  |  |  | 4 RCTs in adults |  |  |  |  |
| Adeniyi, 2012 |  |  |  |  | √ | –2012 | 4 | RCT | Weight management | Non-surgical weight management | No intervention/alternative solution | Asthma Control Test; Medication adherence; PEFR, FEV1 and FVC |
| Jia, 2022 | √ |  |  |  |  | –2021 | 8 | RCT | Supplements | N-3 LC-PUFA | Placebo | Risk of asthma/risk in the offspring |
| Lin, 2020 | √ |  |  |  |  | –2017 | 7 | RCT | Supplements | N-3 LC-PUFA | Usual care | Risk of asthma/risk in the offspring; Incidence of asthma |
| Gunaratne, 2015 | √ |  |  |  |  | 1864–2014 | 8 | RCT | Supplements | N-3 LC-PUFA | Placebo/no intervention | Incidence of asthma |
| Yang, 2015 | √ |  |  |  |  | –2013 | 5 | Case-control study or cohort study | Supplements | Folic acid | NA; Usual care | Risk of asthma/risk in the offspring |
| Colquitt, 2022 | √ |  |  |  |  | 1946–2021 | 6 | RCT | Supplements | Microecological regulator | Placebo | Incidence of asthma |
| Elazab, 2013 | √ |  |  |  |  | 2001–2012 | 25 | RCT | Supplements | Microecological regulator | Placebo | Incidence of asthma |
| Zuccotti, 2015 | √ | √ |  |  |  | –2014 | 17 | RCT | Supplements | Microecological regulator | Placebo | Risk of asthma/risk in the offspring |
| Azad, 2013 | √ | √ |  |  |  | –2013 | 20 | RCT | Supplements | Microecological regulator | Placebo or no intervention | Risk of asthma/risk in the offspring |
| Shen, 2018 | √ | √ |  |  |  | –2017 | 36 | RCT; cohort study | Supplements | Vitamin D | Placebo or no treatment; NA | Risk of asthma/risk in the offspring |
| Li, 2019 | √ |  |  |  |  | –2017 | 7 | RCT or Prospective cohort study | Supplements | Vitamin D | Placebo /no intervention | Risk of asthma/risk in the offspring |
| Yepes-Nunez, 2018 | √ | √ |  |  |  | –2016 | 29 | RCT; NRCT | Supplements | Vitamin D | No intervention | Incidence of asthma |
| Venter, 2020 | √ |  |  |  |  | –2019 | 95 | RCT | Supplements; diet | Dietary pattern; Dietary intake | Placebo /no intervention | Risk of asthma/risk in the offspring |
| Best, 2016 | √ |  |  |  |  | –2015 | 20 | Prospective cohort; RCT | Supplements; diet | N-3 LC-PUFA | NA; Usual care | Risk of asthma/risk in the offspring; Incidence of asthma; Asthma related symptoms |
| Lv, 2014 | √ |  |  |  |  | 1950–2014 | 6 | Cohort study | Diet | Dietary pattern | NA | Risk of asthma/risk in the offspring |
| Netting, 2013 | √ |  |  |  |  | –2011 | 42 | RCT; Cohort; case-control study | Diet | Dietary pattern | No intervention | Risk of asthma/risk in the offspring |
| Zhang, 2019 | √ |  | √ |  |  | –2018 | 7 | Cross-sectional; cohort study | Diet | Dietary pattern | NA | Risk of asthma/risk in the offspring; Incidence of asthma |
| Zairina, 2014 | √ |  |  |  |  | –2013 | 3 | Pre- and post- study; RCT | Others | Complex interventions with multiple behavioral interventions | NA; Placebo; Alternative solution | Medication adherence; Lung function (FEV1, FEV1%, FVC, FEV1, FVC); Asthma related symptoms; Hospitalization; Asthma Control Test |
| Wawryk-Gawda, 2021 |  | √ |  |  |  | 2009–2019 | 11 | RCT | Supplements | Microecological regulator | Placebo | Incidence of asthma |
| Wei, 2020 |  | √ |  |  |  | –2018 | 19 | RCT | Supplements | Microecological regulator | Placebo | Risk of asthma/risk in the offspring |
| Du, 2019 |  | √ | √ |  |  | –2018 | 17 | RCT | Supplements | Microecological regulator | Placebo | Incidence of asthma; Asthma related symptoms |
| Cuello-Garcia, 2017 |  | √ |  |  |  | –2016 | 2 | RCT | Supplements | Microecological regulator | Placebo | Risk of asthma/risk in the offspring |
| Osborn, 2013 |  | √ |  |  |  | –2012 | 4 | Randomized and quasi-randomized controlled trials | Supplements | Microecological regulator | Placebo/no intervention | Risk of asthma/risk in the offspring |
| Osborn, 2018 |  | √ |  |  |  | –2017 | 16 | Randomized and quasi-randomized trials | Supplements | Infant formula | Usual care | Incidence of asthma |
| Luo, 2015 |  |  | √ |  | √ | 1946–2015 | 7 | RCT | Supplements | Vitamin D | NA | Exacerbation; FEV1 |
| Pojsupap, 2015 |  |  | √ | √ |  | –2014 | 5 | RCT | Supplements | Vitamin D | NA | Exacerbation; FEV1 |
| Wang, 2022a^3^ |  |  | √ | √ | √ | –2022 | 19 | RCT | Supplements | Vitamin D | Placebo | FEV1%, FEV1, FVC; Asthma Control Test; Exacerbation; Hospitalization |
| van Brakel, 2020 |  |  | √ | √ | √ | –2019 | 28 | RCT | Supplements | N-3 LC-PUFA  Vitamin D  Antioxidant (vitamin C and E) | NA | Asthma related symptoms |
| Chen, 2021 |  |  | √ |  | √ | –2021 | 12 | RCT | Supplements | Vitamin D | NA | Exacerbation; Asthma Control Test; FEV1% |
| Riverin, 2015 |  |  | √ | √ |  | –2014 | 8 | RCT | Supplements | Vitamin D | Placebo/ alternative solution | Exacerbation; Hospitalization; Emergency visit; Asthma Control Test; Lung function |
| Fares, 2015 |  |  | √ | √ |  | –2013 | 4 | RCT | Supplements | Vitamin D | NA | Asthma related symptoms; FEV1 |
| Jolliffe, 2017 |  |  | √ |  | √ | –2016 | 7 | RCT | Supplements | Vitamin D | NA | Exacerbation; |
| Wang, 2022b^4^ |  |  | √ |  |  | –2020 | 35 | RCT | Supplements | Vitamin D | Placebo | Lung function; Disease control; Incidence of asthma |
| Li, 2022a^5^ |  |  | √ | √ |  | –2022 | 32 | RCT | Supplements | Vitamin D | NA | Risk of asthma/risk in the offspring |
| Abuabat, 2019 |  |  | √ | √ | √ | –2016 | 8 | RCT | Supplements | Magnesium | Placebo /no intervention | FEV1; FVC; Asthma Control Test; |
| Wilkinson, 2014 |  |  | √ |  | √ | –2013 | 5 | RCT | Supplements | Antioxidant (vitamin C and E) | Placebo | Lung function (FEV1, PEF) |
| Lin, 2018 |  |  | √ |  |  | –2017 | 11 | RCT | Supplements | Microecological regulator | NA | Incidence of asthma;Asthma related symptoms; Asthma Control Test; FEV1; PEF |
| Clarke, 2012 |  |  | √ | √ |  | 2000–2010 | 18 | RCT’s; quasi RCT’s or experimental designs | Psychological interventions | Psychological interventions | Usual care | Quality of life |
| Knibb, 2020 |  |  | √ | √ | √ | –2018 | 27 | RCT | Self-management support and health education | Digital health interventions; Education-based interventions delivered by Health professionals; Psychological interventions | NA | Medication adherence; Quality of life; Asthma related symptoms; School absence/days off work |
| Nguyen, 2021 |  |  | √ | √ | √ | 2007–2020 | 6 | RCT; single-arm; nonrandomized; interventional study | Self-management support and health education | Digital health interventions | NA | Medication adherence; Asthma Control Test |
| Farzandipour, 2017 |  |  | √ | √ | √ | 2000–2016 | 10 | RCT; NRCT; observational study | Self-management support and health education | Digital health interventions | NA | Asthma Control Test; Lung function; Quality of life; Hospitalization |
| Kew, 2016a^6^ |  |  | √ | √ | √ | –2016 | 18 | RCT | Self-management support and health education | Digital health interventions; Home-based self-management support | Usual care | Exacerbation; Hospitalization; Asthma Control Test; Quality of life; FEV1% |
| Tran, 2014 |  |  | √ | √ | √ | –2013 | 6 | RCT | Self-management support and health education | Digital health interventions; Home-based self-management support | NA | Medication adherence |
| Ramsey, 2020 |  |  | √ | √ |  | –2019 | 15 | RCT | Self-management support and health education | Digital health interventions | NA | Medication adherence |
| McLean, 2011 |  |  | √ | √ | √ | –2010 | 21 | RCT | Self-management support and health education | Digital health interventions | Usual care | Quality of life; Emergency visit; Hospitalization |
| Culmer, 2020 |  |  | √ | √ |  | –2019 | 5 | RCT; cohort study | Self-management support and health education | Digital health interventions; Education-based interventions delivered by Health professionals | NA | Quality of life |
| Snoswell, 2021 |  | √ | √ | √ | √ | 2000–2018 | 17 | RCT | Self-management support and health education | Digital health interventions | Usual care | Quality of life |
| Morrison, 2014 |  |  | √ | √ | √ | –2011 | 10 | RCT | Self-management support and health education | Digital health interventions | Usual care/ alternative solution | Quality of life; Medication adherence; Asthma related symptoms; School absences; Lung function |
| Hui, 2017 |  |  | √ | √ | √ | 2000–2016 | 12 | RCT and quasi-experimental study | Self-management support and health education | Digital health interventions | No intervention | Disease control |
| Eichenberger, 2013 |  |  | √ |  | √ | –2012 | 17 | RCT | Exercise | Physical exercise | NA | Asthma related symptoms; FEV1%; FEV1, PEF; Quality of Life |
| Yin, 2019 |  |  | √ |  |  | 1990–2017 | 9 | RCT | Exercise | Physical exercise | NA | PEF, FEV1, FVC; Incidence of asthma |
| Beggs, 2013 |  |  | √ | √ |  | –2012 | 8 | RCT, quasi-RCT | Exercise | Physical exercise | Usual care/ alternative solution | Quality of life; Asthma related symptoms; Medication adherence; FEV1%, FVC, PEF |
| Zhu, 2022 |  |  | √ | √ | √ | –2021 | 18 | RCT | Exercise | Physical exercise | Alternative solution | FEV1 %; Asthma Control Test |
| Ramachandran, 2021 |  |  | √ |  |  | –2020 | 9 | RCT; two-arm quasi-experimental study; three-arm quasi-experimental study; four-arm quasi-experimental study; two-arm interventional study with a control group | Exercise | Physical exercise | No intervention, usual care or alternative solution | FEV1%, FEV1, FVC, PEF; Medication adherence; Asthma related symptoms |
| Li, 2022b^7^ |  |  | √ | √ | √ | 2000–2021 | 14 | RCT | Exercise | Physical exercise | Usual care | FEV1, FVC, PEF |
| Liu, 2021 |  |  | √ |  |  | –2021 | 22 | RCT | Exercise | Physical exercise | Usual care | FVC, PEF, FEV1; Quality of life |
| Wanrooij, 2014 |  |  | √ | √ |  | –2012 | 29 | Controlled trials | Exercise | Physical exercise | NA | Lung function; Quality of life; Disease control |
| Crosbie, 2012 |  |  | √ |  |  | –2011 | 16 | RCT | Exercise | Physical exercise | Usual care | Lung function; Quality of Life |
| Jiang, 2022 |  |  | √ |  |  | –2021 | 24 | RCT | Exercise | Physical exercise | Usual care | FEV1%, FEF 25%~75% |
| Pacheco, 2012 |  |  | √ | √ | √ | 2000–2010 | 11 | RCT | Exercise | Physical exercise; Breathing exercise | Usual care | Quality of life |
| Ertürk, 2022 |  |  |  | √ | √ | 2000–2021 | 7 | RCT | Exercise | Physical exercise | Alternative solution/ no intervention | Lung function |
| Tyson, 2022 |  |  |  |  | √ | 1990–2020 | 25 | RCT, non-randomized controlled trials, before-and-after study, a quasi-experimental study, and a feasibility study | Exercise | Physical exercise | NA | Quality of life; Asthma Control Test; Asthma related symptoms; Medication adherence |
| Kuder, 2021 |  |  |  |  | √ | –2019 | 35 | RCT | Exercise | Physical exercise | NA | Lung function; Disease control;  Quality of life |
| McLoughlin, 2022 |  |  |  |  | √ | –2021 | 4 | RCT | Exercise | Physical exercise | NA | Disease control; Quality of life; Lung function |

| Hansen, 2020 |  |  |  |  | √ | –2019 | 11 | RCT | Exercise | Physical exercise | NA | Disease control; Lung function |
| --- | --- | --- | --- | --- | --- | --- | --- | --- | --- | --- | --- | --- |
| Ang, 2022 |  |  |  |  | √ | 2012–2022 | 14 | RCT; prospective study | Exercise | Physical exercise | NA | Disease control; Quality of life |
| Feng, 2021 |  |  |  |  | √ | –2019 | 9 | RCT | Exercise | Physical exercise | NA | Quality of life; Asthma Control Test; Asthma related symptoms; FEV1; FVC; PEF |
| Heikkinen, 2012 |  |  |  |  | √ | 1980–2011 | 11 | RCT; NRCT | Exercise | Physical exercise | NA | FEV1; Quality of life; Asthma Control Test |
| Osadnik, 2022 |  |  |  |  | √ | –2021 | 10 | RCT | Exercise | Breathing exercise | Usual care | Asthma Control Test; Quality of life |
| Grande, 2014 |  |  |  |  | √ | –2014 | 3 | RCT | Exercise | Physical exercise | Usual care; Physical exercise; non-exercise | Exacerbation; FEV1 |
| Macêdo, 2016 |  |  | √ |  |  | –2015 | 3 | RCT | Exercise | Breathing exercise | placebo; Education-based interventions delivered by Health professionals | Quality of life; Asthma related symptoms |
| Zhang, 2021 |  |  | √ |  |  | –2018 | 18 | RCT | Exercise | Physical exercise；Breathing exercise | Usual care | PEF; FEV1; FVC |
| Das, 2019 |  |  | √ |  |  | –2018 | 10 | RCT | Exercise | Breathing exercise | NA | Quality of life; Asthma related symptoms; Exacerbation; Lung function |
| Burgess, 2011 |  |  | √ | √ | √ | 1954–2011 | 41 | RCT | Exercise | Breathing exercise | NA | PEF; FEV1; Quality of life; Lung function; Asthma related symptoms |
| Freitas, 2013 |  |  |  |  | √ | –2013 | 13 | RCT | Exercise | Breathing exercise | no intervention | Asthma related symptoms; Quality of life; Exacerbation; FEV1 |
| Santino, 2020 |  |  |  |  | √ | –2019 | 22 | RCT | Exercise | Breathing exercise | no intervention; Self-management support and health education | Quality of life; Asthma related symptoms; FEV1%; FEV1 |
| Prem, 2013 |  |  |  |  | √ | –2011 | 3 | RCT | Exercise | Breathing exercise | alternative solution；Self-management support and health education | Quality of life; Lung function |
| Lista-Paz, 2022 |  |  |  |  | √ | –2021 | 11 | Randomized controlled trials and quasi-experimental study | Exercise | Breathing exercise | NA | FEV1; FVC |
| Silva, 2013 |  |  |  |  | √ | –2012 | 5 | RCT | Exercise | Breathing exercise | placebo | Lung function; Asthma related symptoms |
| Marcano Belisario, 2013 |  |  |  | √ | √ | 2000–2013 | 2 | RCT | Self-management support and health education | Digital health interventions | alternative solution | Asthma related symptoms; Quality of life; Emergency visit; Hospitalization |
| Alquran, 2018 |  |  |  | √ |  | 2007–2018 | 8 | No restriction | Self-management support and health education | Digital health interventions | NA | Disease control; Medication adherence |
| McLean, 2016 |  |  |  |  | √ | –2014 | 8 | RCT | Self-management support and health education | Digital health interventions | Usual care | Quality of life; Disease control; |
| Schulte, 2021 |  |  |  |  | √ | 2000–2020 | 7 | RCT | Self-management support and health education | Digital health interventions | Usual care; alternative solution | Medication adherence |
| Paudyal, 2018 |  |  |  |  | √ | –2016 | 4 | RCT | Psychological interventions | Psychological interventions | NA | Quality of life; FEV1; PEF |
| Yorke, 2015 |  |  |  |  | √ | –2013 | 23 | RCT | Others | Complex interventions with multiple behavioral interventions | NA; self-management support and health education | FEV1; Medication adherence; Quality of life; Asthma related symptoms |
| Miller, 2017 |  |  | √ | √ | √ | –2016 | 11 | RCT | Self-management support and health education | Digital health interventions | Usual care; alternative solution | Medication adherence; Asthma related symptoms |
| Paudyal, 2014 |  |  |  | √ | √ | –2014 | 4 | RCT | Psychological interventions | Psychological interventions | alternative solution | FVC; Asthma related symptoms; Disease control; Quality of life |
| Wu, 2020 |  |  | √ | √ | √ | –2019 | 22 | RCT | Exercise | Physical exercise | no intervention; Self-management support and health education; alternative solution | FEV1; PEF; FVC; FEV1/FVC%; Quality of life |
| Kew, 2016b^8^ |  |  |  |  | √ | –2016 | 9 | RCT | Psychological interventions | Psychological interventions | Usual care | Quality of life; Exacerbation; Disease control; Medication adherence |

**Abbreviations:** SR/MA=systematic review or meta-analysis; NA=not applicable; CI=confidence interval; OR=odds ratio; RR=relative risk; SMD=standardized mean difference; WMD=weighted mean difference; MD=mean difference; RCT= randomized controlled trials; CBT= Cognitive behavioral therapy; ED=emergency department; ACT=Asthma Control Test; ACQ=Asthma Control Questionnaire; QoL=quality of life; AQLQ=Asthma Quality of Life Questionnaire; PAQLQ=Pediatric Asthma Quality of Life Questionnaire; SGRQ=St. George's Respiratory Questionnaire; HRQOL=health-related quality of life; VD=vitamin D; FeNO=fraction of exhaled Nitric Oxide; FEV1=forced expiratory volume in one second; FVC=forced vital capacity; PEF=peak expiratory flow; PEFR=peak expiratory flow rate; FEF 25%~75%=forced expiratory flow at 25-75%; VO2 max=maximal oxygen consumption.

**References:**

1. Kew KM, Malik P, Aniruddhan K, Normansell R. Shared decision-making for people with asthma. The Cochrane Database of Systematic Reviews. 2017;10(10):CD012330.

2. Kew KM, Carr R, Crossingham I. Lay-led and peer support interventions for adolescents with asthma. The Cochrane Database of Systematic Reviews. 2017;4(4):CD012331.

3. Wang Y, Wang J, Chen L, Zhang H, Yu L, Chi Y, et al. Efficacy of vitamin D supplementation on COPD and asthma control: A systematic review and meta-analysis. Journal of Global Health. 2022;12:04100.

4. Wang Q, Ying Q, Zhu W, Chen J. Vitamin D and asthma occurrence in children: A systematic review and meta-analysis. Journal of Pediatric Nursing. 2022;62:e60-e8.

5. Li Q, Zhou Q, Zhang G, Tian X, Li Y, Wang Z, et al. Vitamin D Supplementation and Allergic Diseases during Childhood: A Systematic Review and Meta-Analysis. Nutrients. 2022;14(19).

6. Kew KM, Cates CJ. Home telemonitoring and remote feedback between clinic visits for asthma. The Cochrane Database of Systematic Reviews. 2016;2016(8):CD011714.

7. Li X, Mao C, Pan Y. Effect of Routine Therapy Assisted by Physical Exercise on Pulmonary Function in Patients with Asthma in Stable Stage: A Systematic Review and Meta-analysis of Randomized Clinical Trials. Comput Math Methods Med. 2022;2022:2350297.

8. Kew KM, Nashed M, Dulay V, Yorke J. Cognitive behavioural therapy (CBT) for adults and adolescents with asthma. The Cochrane Database of Systematic Reviews. 2016;9(9):CD011818.
