# Supplementary material for: Non‐pharmacological interventions for asthma prevention and management across the life course: Umbrella review
Source: Clin Transl Allergy. 2024 Feb 29;14(3):e12344. doi: 10.1002/clt2.12344 (PMC10904350; doi:10.1002/clt2.12344)
Supplement: Supplementary file 4 — Table S4 [file CLT2-14-e12344-s003.docx]

**Table S4 Item-by-item quality rating using the AMSTAR-2.**

| **AMSTAR-2 item^*^** | **1** | **2** | **3** | **4** | **5** | **6** | **7** | **8** | **9** | **10** | **11** | **12** | **13** | **14** | **15** | **16** | **Overall confidence** |
| --- | --- | --- | --- | --- | --- | --- | --- | --- | --- | --- | --- | --- | --- | --- | --- | --- | --- |
| Azad, 2013 | 1 | 2 | 1 | 1 | 1 | 1 | 0 | 2 | 3 | 1 | 2 | 2 | 1 | 1 | 1 | 1 | Critically low |
| Abuabat, 2019 | 1 | 1 | 0 | 1 | 1 | 1 | 0 | 2 | 3 | 0 | 2 | 1 | 0 | 1 | 0 | 1 | Critically low |
| Adeniyi, 2012 | 1 | 0 | 1 | 2 | 1 | 1 | 1 | 2 | 3 | 1 | 0 | 0 | 0 | 1 | 0 | 1 | Critically low |
| Ahmad, 2011 | 1 | 0 | 1 | 1 | 0 | 0 | 2 | 1 | 0 | 0 | 0 | 0 | 0 | 0 | 0 | 0 | Critically low |
| Ahmed, 2018 | 1 | 2 | 1 | 1 | 1 | 1 | 0 | 2 | 2 | 1 | 0 | 0 | 1 | 1 | 0 | 1 | Critically low |
| Alquran, 2018 | 0 | 0 | 1 | 1 | 1 | 1 | 0 | 2 | 1 | 1 | 0 | 0 | 0 | 0 | 0 | 1 | Critically low |
| Ang, 2022 | 1 | 0 | 0 | 1 | 1 | 1 | 0 | 2 | 3 | 0 | 0 | 0 | 0 | 0 | 0 | 1 | Critically low |
| Azad, 2013 | 1 | 2 | 1 | 1 | 1 | 1 | 0 | 2 | 3 | 1 | 2 | 2 | 1 | 1 | 1 | 1 | Critically low |
| Beggs, 2013 | 1 | 2 | 1 | 2 | 1 | 1 | 1 | 2 | 3 | 1 | 2 | 2 | 1 | 1 | 1 | 1 | Low |
| Best, 2016 | 1 | 0 | 0 | 1 | 0 | 0 | 0 | 2 | 3 | 1 | 2 | 1 | 1 | 1 | 1 | 1 | Critically low |
| Burgess, 2011 | 1 | 1 | 0 | 1 | 1 | 1 | 0 | 2 | 1 | 0 | 2 | 1 | 1 | 1 | 1 | 1 | Critically low |
| Carson, 2013 | 1 | 2 | 0 | 0 | 1 | 1 | 1 | 2 | 3 | 0 | 2 | 2 | 1 | 1 | 1 | 1 | Critically low |
| Carvalho Coelho, 2016 | 1 | 0 | 1 | 1 | 1 | 1 | 0 | 2 | 1 | 0 | 0 | 0 | 0 | 0 | 0 | 1 | Critically low |
| Chan, 2021 | 1 | 2 | 1 | 1 | 0 | 0 | 0 | 2 | 3 | 1 | 2 | 2 | 1 | 1 | 2 | 1 | Critically low |
| Chen, 2021 | 1 | 1 | 0 | 1 | 1 | 1 | 0 | 2 | 3 | 0 | 2 | 1 | 1 | 1 | 2 | 1 | Critically low |
| Clarke, 2012 | 1 | 0 | 1 | 1 | 1 | 1 | 0 | 2 | 1 | 0 | 0 | 0 | 0 | 0 | 0 | 0 | Critically low |
| Colquitt, 2022 | 1 | 0 | 1 | 1 | 0 | 0 | 0 | 2 | 3 | 1 | 0 | 0 | 1 | 0 | 0 | 1 | Critically low |
| Crocker, 2011 | 1 | 0 | 1 | 1 | 1 | 1 | 0 | 1 | 1 | 1 | 0 | 0 | 0 | 0 | 1 | 1 | Critically low |
| Crosbie, 2012 | 1 | 1 | 0 | 1 | 0 | 0 | 0 | 2 | 1 | 0 | 0 | 0 | 0 | 0 | 0 | 1 | Critically low |
| Cuello-Garcia, 2017 | 1 | 1 | 0 | 1 | 1 | 1 | 1 | 2 | 3 | 1 | 2 | 1 | 1 | 1 | 2 | 1 | Critically low |
| Culmer, 2020 | 1 | 1 | 0 | 1 | 1 | 1 | 0 | 2 | 2 | 0 | 0 | 0 | 1 | 0 | 0 | 1 | Critically low |
| Das, 2019 | 1 | 1 | 0 | 1 | 1 | 1 | 0 | 2 | 3 | 1 | 2 | 1 | 1 | 1 | 2 | 1 | Critically low |
| deGroene, 2011 | 1 | 2 | 1 | 2 | 1 | 1 | 1 | 2 | 1 | 1 | 2 | 2 | 1 | 1 | 2 | 1 | Low |
| Denford, 2014 | 1 | 0 | 1 | 1 | 1 | 1 | 0 | 2 | 3 | 1 | 2 | 2 | 1 | 1 | 1 | 0 | Critically low |
| Dogaru, 2014 | 1 | 0 | 0 | 1 | 1 | 1 | 0 | 2 | 3 | 0 | 2 | 1 | 1 | 1 | 2 | 1 | Critically low |
| Dokbua, 2018 | 0 | 0 | 1 | 1 | 1 | 1 | 0 | 2 | 2 | 1 | 2 | 2 | 1 | 1 | 1 | 1 | Critically low |
| Du, 2019 | 1 | 1 | 0 | 0 | 1 | 1 | 0 | 2 | 3 | 0 | 2 | 1 | 1 | 1 | 1 | 1 | Critically low |
| Eichenberger, 2013 | 1 | 0 | 1 | 1 | 1 | 1 | 0 | 2 | 3 | 1 | 2 | 2 | 1 | 1 | 1 | 1 | Critically low |
| Elazab, 2013 | 1 | 2 | 1 | 1 | 1 | 1 | 1 | 2 | 3 | 1 | 2 | 2 | 1 | 1 | 2 | 1 | Low |
| Ertürk, 2022 | 1 | 0 | 0 | 1 | 1 | 1 | 1 | 2 | 3 | 0 | 0 | 0 | 0 | 0 | 0 | 1 | Critically low |
| Fares, 2015 | 1 | 2 | 0 | 1 | 1 | 1 | 0 | 2 | 3 | 0 | 0 | 0 | 0 | 1 | 0 | 1 | Critically low |
| Farzandipour, 2017 | 0 | 0 | 1 | 1 | 1 | 1 | 0 | 2 | 1 | 1 | 0 | 0 | 0 | 0 | 0 | 1 | Critically low |
| Feng, 2021 | 1 | 0 | 0 | 1 | 1 | 1 | 0 | 2 | 3 | 0 | 2 | 2 | 1 | 1 | 2 | 1 | Critically low |
| Fidler, 2021 | 1 | 0 | 1 | 1 | 1 | 1 | 0 | 2 | 3 | 1 | 2 | 2 | 1 | 1 | 2 | 1 | Critically low |
| Forte, 2018 | 0 | 2 | 1 | 1 | 1 | 1 | 0 | 2 | 3 | 0 | 0 | 0 | 1 | 1 | 0 | 0 | Critically low |
| Freitas, 2013 | 1 | 2 | 0 | 2 | 1 | 1 | 1 | 2 | 3 | 0 | 2 | 2 | 1 | 1 | 1 | 1 | Low |
| Garagorri-Gutierrez, 2022 | 1 | 0 | 1 | 1 | 0 | 0 | 0 | 2 | 1 | 0 | 0 | 0 | 0 | 0 | 0 | 1 | Critically low |
| Gatheral, 2017 | 1 | 2 | 1 | 2 | 1 | 1 | 1 | 2 | 3 | 1 | 2 | 2 | 1 | 1 | 1 | 1 | Low |
| Gesinde, 2018 | 0 | 0 | 1 | 1 | 0 | 0 | 0 | 2 | 1 | 0 | 0 | 0 | 0 | 0 | 0 | 0 | Critically low |
| Grande, 2014 | 1 | 2 | 0 | 2 | 1 | 1 | 1 | 2 | 3 | 1 | 0 | 0 | 1 | 1 | 0 | 1 | Critically low |
| Gunaratne, 2015 | 1 | 2 | 1 | 1 | 1 | 1 | 1 | 2 | 3 | 1 | 2 | 2 | 1 | 1 | 1 | 1 | Critically low |
| Gungor, 2019 | 1 | 0 | 0 | 1 | 1 | 1 | 1 | 2 | 3 | 1 | 0 | 0 | 1 | 0 | 0 | 1 | Critically low |
| Hansen, 2020 | 1 | 2 | 0 | 1 | 1 | 1 | 0 | 2 | 3 | 0 | 2 | 1 | 1 | 1 | 1 | 1 | Critically low |
| Harris, 2019 | 1 | 2 | 1 | 1 | 1 | 1 | 1 | 2 | 1 | 1 | 2 | 2 | 1 | 1 | 2 | 1 | Critically low |
| Heikkinen, 2012 | 1 | 0 | 0 | 1 | 1 | 1 | 0 | 2 | 1 | 0 | 2 | 1 | 1 | 1 | 0 | 1 | Critically low |
| Henneberger, 2019 | 1 | 2 | 1 | 1 | 1 | 1 | 0 | 2 | 3 | 1 | 2 | 2 | 1 | 1 | 2 | 1 | Critically low |
| Henneberger, 2021 | 1 | 0 | 1 | 1 | 1 | 1 | 0 | 2 | 1 | 1 | 2 | 1 | 0 | 1 | 1 | 1 | Critically low |
| Hodkinson, 2020 | 0 | 0 | 1 | 1 | 1 | 1 | 0 | 2 | 3 | 1 | 2 | 2 | 1 | 1 | 2 | 1 | Critically low |
| Hossain, 2021 | 1 | 0 | 1 | 1 | 1 | 1 | 0 | 2 | 1 | 0 | 0 | 0 | 0 | 0 | 0 | 0 | Critically low |
| Hosseini, 2017 | 1 | 0 | 1 | 1 | 1 | 1 | 0 | 2 | 1 | 0 | 1 | 1 | 0 | 1 | 1 | 1 | Critically low |
| Hu, 2022 | 1 | 2 | 1 | 1 | 1 | 1 | 0 | 2 | 1 | 1 | 0 | 0 | 0 | 0 | 0 | 1 | Critically low |
| Hui, 2017 | 1 | 2 | 1 | 1 | 1 | 1 | 0 | 2 | 3 | 1 | 0 | 0 | 1 | 0 | 0 | 1 | Critically low |
| Isik, 2019 | 1 | 0 | 1 | 1 | 0 | 0 | 0 | 2 | 3 | 0 | 0 | 0 | 0 | 0 | 0 | 0 | Critically low |
| Jia, 2022 | 1 | 2 | 1 | 0 | 1 | 1 | 0 | 2 | 3 | 1 | 2 | 2 | 1 | 1 | 2 | 1 | Critically low |
| Jiang, 2022 | 1 | 2 | 0 | 1 | 1 | 1 | 0 | 2 | 3 | 0 | 2 | 1 | 0 | 1 | 1 | 1 | Critically low |
| Jolliffe, 2017 | 1 | 2 | 0 | 1 | 1 | 1 | 0 | 2 | 3 | 0 | 2 | 1 | 1 | 1 | 2 | 1 | Critically low |
| Juel, 2012 | 0 | 0 | 1 | 1 | 0 | 0 | 0 | 1 | 1 | 0 | 0 | 0 | 0 | 0 | 0 | 1 | Critically low |
| Kew, 2016 a^1^ | 1 | 2 | 0 | 2 | 1 | 1 | 1 | 2 | 3 | 1 | 2 | 2 | 1 | 1 | 2 | 1 | High |
| Kew, 2016 b^2^ | 1 | 2 | 0 | 2 | 1 | 1 | 1 | 2 | 3 | 1 | 2 | 2 | 1 | 1 | 1 | 1 | Low |
| Kew, 2017 a^3^ | 1 | 2 | 1 | 1 | 1 | 1 | 1 | 2 | 3 | 1 | 0 | 0 | 1 | 1 | 0 | 1 | Critically low |
| Kew, 2017 b^4^ | 1 | 2 | 0 | 2 | 1 | 1 | 1 | 2 | 3 | 1 | 2 | 2 | 1 | 1 | 1 | 1 | Low |
| Khalooeifard, 2021 | 1 | 0 | 1 | 1 | 1 | 1 | 0 | 2 | 1 | 1 | 2 | 2 | 1 | 1 | 2 | 1 | Critically low |
| Kneale, 2019 | 0 | 2 | 1 | 1 | 1 | 1 | 0 | 2 | 3 | 1 | 2 | 2 | 1 | 1 | 2 | 1 | Critically low |
| Knibb, 2020 | 1 | 2 | 1 | 1 | 1 | 1 | 0 | 2 | 3 | 1 | 0 | 0 | 1 | 0 | 0 | 1 | Critically low |
| Kuder, 2021 | 1 | 1 | 0 | 2 | 1 | 1 | 1 | 2 | 3 | 0 | 0 | 0 | 1 | 0 | 0 | 1 | Critically low |
| Leas, 2018 | 1 | 0 | 1 | 1 | 1 | 1 | 0 | 2 | 3 | 1 | 0 | 0 | 0 | 1 | 2 | 1 | Critically low |
| Lee, 2021 | 1 | 2 | 1 | 1 | 0 | 0 | 0 | 2 | 3 | 1 | 0 | 0 | 0 | 0 | 0 | 1 | Critically low |
| Li, 2019 | 1 | 2 | 1 | 1 | 1 | 1 | 0 | 2 | 3 | 1 | 2 | 2 | 1 | 1 | 2 | 1 | Critically low |
| Li, 2022 a^5^ | 1 | 2 | 1 | 1 | 1 | 1 | 0 | 2 | 3 | 1 | 2 | 2 | 1 | 1 | 2 | 1 | Low |
| Li, 2022 b^6^ | 1 | 0 | 0 | 1 | 1 | 1 | 0 | 2 | 0 | 0 | 2 | 1 | 0 | 1 | 1 | 1 | Critically low |
| Liao, 2019 | 1 | 2 | 1 | 1 | 1 | 1 | 0 | 2 | 1 | 1 | 0 | 0 | 0 | 0 | 0 | 1 | Critically low |
| Lin, 2018 | 0 | 0 | 1 | 1 | 1 | 1 | 0 | 2 | 3 | 0 | 2 | 2 | 1 | 1 | 1 | 1 | Critically low |
| Lin, 2020 | 1 | 0 | 1 | 1 | 1 | 1 | 0 | 2 | 3 | 1 | 2 | 2 | 1 | 1 | 2 | 1 | Critically low |
| Lista-Paz, 2022 | 1 | 1 | 0 | 1 | 1 | 1 | 0 | 2 | 3 | 1 | 2 | 2 | 1 | 1 | 2 | 1 | Critically low |
| Liu, 2021 | 1 | 0 | 0 | 1 | 1 | 1 | 0 | 2 | 3 | 0 | 2 | 1 | 1 | 1 | 2 | 1 | Critically low |
| Luo, 2015 | 1 | 1 | 0 | 1 | 1 | 1 | 0 | 2 | 3 | 0 | 2 | 1 | 0 | 1 | 2 | 1 | Critically low |
| Lv, 2014 | 1 | 0 | 0 | 1 | 1 | 1 | 0 | 2 | 1 | 0 | 2 | 2 | 1 | 1 | 2 | 1 | Critically low |
| Lv, 2015 | 1 | 2 | 1 | 1 | 1 | 1 | 0 | 2 | 1 | 1 | 0 | 0 | 0 | 0 | 0 | 1 | Critically low |
| Macêdo, 2016 | 1 | 2 | 0 | 2 | 1 | 1 | 0 | 2 | 3 | 1 | 0 | 0 | 1 | 1 | 0 | 1 | Critically low |
| Marcano Belisario, 2013 | 1 | 2 | 1 | 1 | 1 | 1 | 1 | 2 | 1 | 0 | 0 | 0 | 1 | 1 | 0 | 1 | Critically low |
| Maricoto, 2019 | 1 | 2 | 1 | 1 | 1 | 1 | 0 | 2 | 3 | 1 | 2 | 2 | 1 | 1 | 1 | 1 | Critically low |
| McCallum, 2017 | 1 | 2 | 1 | 1 | 1 | 1 | 1 | 2 | 3 | 1 | 2 | 2 | 1 | 1 | 1 | 1 | Critically low |
| McLean, 2011 | 1 | 1 | 0 | 2 | 1 | 1 | 0 | 2 | 3 | 0 | 2 | 2 | 1 | 1 | 1 | 1 | Critically low |
| McLean, 2016 | 1 | 2 | 1 | 1 | 1 | 1 | 0 | 2 | 3 | 1 | 2 | 1 | 0 | 0 | 2 | 1 | Critically low |
| McLoughlin, 2022 | 1 | 1 | 0 | 1 | 1 | 1 | 0 | 2 | 3 | 0 | 2 | 1 | 1 | 1 | 1 | 1 | Critically low |
| Miller, 2017 | 1 | 0 | 0 | 2 | 1 | 1 | 0 | 2 | 3 | 0 | 2 | 2 | 1 | 1 | 2 | 1 | Critically low |
| Morrison, 2014 | 1 | 2 | 1 | 2 | 1 | 1 | 1 | 2 | 1 | 1 | 0 | 0 | 0 | 0 | 0 | 1 | Critically low |
| Mosnaim, 2016 | 1 | 0 | 0 | 1 | 1 | 1 | 0 | 2 | 1 | 0 | 0 | 0 | 0 | 0 | 0 | 1 | Critically low |
| Mosnaim, 2017 | 0 | 0 | 0 | 1 | 1 | 1 | 0 | 2 | 1 | 1 | 0 | 0 | 0 | 0 | 0 | 1 | Critically low |
| Netting, 2013 | 1 | 0 | 1 | 2 | 1 | 1 | 1 | 2 | 3 | 0 | 2 | 2 | 1 | 1 | 1 | 1 | Critically low |
| Nguyen, 2021 | 1 | 1 | 0 | 1 | 1 | 1 | 0 | 2 | 3 | 0 | 0 | 0 | 1 | 0 | 0 | 1 | Critically low |
| Normansell, 2017 | 1 | 2 | 0 | 2 | 1 | 1 | 0 | 2 | 3 | 1 | 2 | 2 | 1 | 1 | 2 | 1 | Low |
| Nurmatov, 2011 | 1 | 1 | 0 | 2 | 1 | 1 | 0 | 2 | 3 | 0 | 2 | 2 | 1 | 1 | 1 | 1 | Critically low |
| Okoniewski, 2019 | 1 | 2 | 1 | 1 | 1 | 1 | 0 | 2 | 3 | 1 | 0 | 0 | 0 | 0 | 0 | 1 | Critically low |
| Osadnik, 2022 | 1 | 2 | 0 | 2 | 1 | 1 | 1 | 2 | 3 | 1 | 2 | 1 | 1 | 1 | 1 | 1 | Low |
| Osborn, 2013 | 1 | 1 | 0 | 2 | 1 | 1 | 1 | 2 | 3 | 1 | 2 | 1 | 1 | 1 | 2 | 1 | Low |
| Osborn, 2018 | 1 | 2 | 0 | 2 | 1 | 1 | 1 | 2 | 3 | 1 | 2 | 1 | 1 | 1 | 2 | 1 | Moderate |
| Pacheco, 2012 | 1 | 1 | 0 | 1 | 1 | 1 | 0 | 2 | 3 | 0 | 0 | 0 | 0 | 1 | 0 | 1 | Critically low |
| Paudyal, 2014 | 1 | 1 | 0 | 2 | 1 | 1 | 1 | 2 | 3 | 1 | 2 | 2 | 1 | 1 | 2 | 1 | Low |
| Paudyal, 2018 | 1 | 1 | 0 | 1 | 1 | 1 | 0 | 2 | 3 | 0 | 2 | 2 | 1 | 1 | 1 | 1 | Critically low |
| Peytremann-Bridevaux, 2015 | 1 | 2 | 1 | 1 | 1 | 1 | 1 | 2 | 1 | 1 | 0 | 0 | 1 | 1 | 0 | 1 | Critically low |
| Pinnock, 2015 | 1 | 2 | 1 | 1 | 1 | 1 | 0 | 2 | 1 | 1 | 0 | 0 | 0 | 1 | 0 | 1 | Critically low |
| Pinnock, 2017 | 1 | 2 | 1 | 1 | 1 | 1 | 0 | 2 | 3 | 1 | 0 | 0 | 1 | 1 | 0 | 1 | Critically low |
| Pogson, 2011 | 1 | 2 | 1 | 1 | 1 | 1 | 1 | 2 | 3 | 1 | 0 | 0 | 1 | 0 | 0 | 1 | Critically low |
| Pojsupap, 2015 | 1 | 1 | 0 | 1 | 1 | 1 | 0 | 2 | 3 | 0 | 2 | 2 | 1 | 1 | 1 | 1 | Critically low |
| Prem, 2013 | 1 | 0 | 0 | 1 | 1 | 1 | 0 | 2 | 3 | 0 | 0 | 0 | 0 | 0 | 0 | 1 | Critically low |
| Press, 2012 | 1 | 0 | 1 | 1 | 1 | 1 | 0 | 2 | 3 | 1 | 0 | 0 | 0 | 1 | 0 | 1 | Critically low |
| Ramachandran, 2021 | 1 | 1 | 0 | 2 | 1 | 1 | 1 | 2 | 3 | 0 | 2 | 1 | 1 | 1 | 1 | 1 | Low |
| Ramdzan, 2021 | 1 | 2 | 1 | 1 | 1 | 1 | 0 | 2 | 2 | 1 | 0 | 0 | 1 | 0 | 0 | 1 | Critically low |
| Ramsey, 2020 | 1 | 1 | 0 | 1 | 1 | 1 | 0 | 2 | 1 | 0 | 0 | 0 | 1 | 0 | 0 | 1 | Critically low |
| Riverin, 2015 | 1 | 1 | 0 | 1 | 1 | 1 | 0 | 2 | 3 | 0 | 2 | 1 | 1 | 1 | 2 | 1 | Critically low |
| Santino, 2021 | 1 | 2 | 0 | 2 | 1 | 1 | 1 | 2 | 3 | 1 | 2 | 2 | 1 | 1 | 2 | 1 | High |
| Schuers, 2019 | 1 | 0 | 1 | 1 | 1 | 1 | 0 | 2 | 1 | 0 | 0 | 0 | 0 | 0 | 0 | 1 | Critically low |
| Schulte, 2021 | 1 | 0 | 1 | 1 | 1 | 1 | 0 | 2 | 3 | 1 | 0 | 0 | 1 | 0 | 0 | 1 | Critically low |
| Shen, 2018 | 1 | 2 | 1 | 1 | 1 | 1 | 1 | 2 | 3 | 1 | 2 | 2 | 1 | 1 | 2 | 1 | Low |
| Silva, 2013 | 1 | 2 | 0 | 1 | 1 | 1 | 1 | 2 | 3 | 0 | 2 | 1 | 1 | 1 | 1 | 1 | Critically low |
| Snoswell, 2021 | 1 | 2 | 1 | 1 | 1 | 1 | 0 | 2 | 1 | 1 | 2 | 1 | 0 | 1 | 2 | 1 | Critically low |
| Song, 2021 | 1 | 2 | 1 | 1 | 1 | 1 | 0 | 2 | 3 | 1 | 2 | 2 | 1 | 1 | 1 | 1 | Critically low |
| Tran, 2014 | 1 | 1 | 0 | 1 | 1 | 1 | 0 | 2 | 1 | 0 | 0 | 0 | 1 | 0 | 0 | 1 | Critically low |
| Tyson, 2022 | 1 | 1 | 0 | 1 | 1 | 1 | 0 | 2 | 3 | 0 | 0 | 0 | 0 | 0 | 0 | 1 | Critically low |
| Uchima, 2019 | 1 | 0 | 1 | 1 | 0 | 0 | 0 | 2 | 3 | 0 | 0 | 0 | 0 | 0 | 0 | 0 | Critically low |
| Upala, 2019 | 1 | 2 | 1 | 1 | 1 | 1 | 0 | 2 | 2 | 0 | 2 | 1 | 0 | 0 | 1 | 1 | Critically low |
| van Brakel, 2020 | 1 | 1 | 0 | 1 | 1 | 1 | 0 | 2 | 2 | 0 | 0 | 0 | 1 | 1 | 0 | 1 | Critically low |
| Venter, 2020 | 1 | 0 | 1 | 1 | 1 | 1 | 0 | 2 | 3 | 1 | 2 | 2 | 1 | 1 | 2 | 1 | Critically low |
| Villa-Roel, 2016 | 1 | 2 | 1 | 1 | 1 | 1 | 0 | 2 | 3 | 1 | 2 | 1 | 0 | 1 | 1 | 1 | Critically low |
| Walter, 2016 | 1 | 0 | 1 | 1 | 1 | 1 | 1 | 2 | 3 | 0 | 0 | 0 | 0 | 0 | 0 | 1 | Critically low |
| Wang, 2022 a^7^ | 1 | 2 | 0 | 1 | 1 | 1 | 0 | 2 | 3 | 0 | 2 | 1 | 1 | 1 | 1 | 1 | Critically low |
| Wang, 2022 b^8^ | 0 | 0 | 1 | 1 | 1 | 1 | 0 | 2 | 1 | 1 | 2 | 1 | 0 | 1 | 1 | 1 | Critically low |
| Wanrooij, 2014 | 1 | 1 | 0 | 1 | 1 | 1 | 0 | 2 | 1 | 0 | 0 | 0 | 0 | 0 | 0 | 1 | Critically low |
| Wawryk-Gawda, 2021 | 1 | 1 | 0 | 1 | 1 | 1 | 0 | 2 | 3 | 0 | 2 | 1 | 1 | 1 | 2 | 1 | Critically low |
| Wei, 2020 | 1 | 0 | 0 | 1 | 1 | 1 | 0 | 2 | 3 | 0 | 2 | 1 | 1 | 1 | 2 | 1 | Critically low |
| Welker, 2018 | 1 | 0 | 1 | 1 | 1 | 1 | 0 | 2 | 1 | 0 | 0 | 0 | 0 | 0 | 0 | 1 | Critically low |
| Welsh, 2011 | 1 | 2 | 1 | 1 | 1 | 1 | 1 | 2 | 3 | 1 | 0 | 0 | 1 | 1 | 0 | 1 | Critically low |
| Wilkinson, 2014 | 1 | 2 | 0 | 1 | 1 | 1 | 1 | 2 | 3 | 1 | 0 | 0 | 1 | 1 | 2 | 1 | Critically low |
| Wu, 2020 | 1 | 0 | 0 | 0 | 1 | 1 | 0 | 2 | 3 | 0 | 2 | 2 | 1 | 1 | 2 | 1 | Critically low |
| Xue, 2021 | 1 | 2 | 0 | 1 | 1 | 1 | 0 | 2 | 3 | 0 | 2 | 2 | 1 | 1 | 2 | 1 | Critically low |
| Yang, 2015 | 1 | 0 | 1 | 1 | 1 | 1 | 0 | 2 | 3 | 0 | 2 | 2 | 1 | 1 | 2 | 1 | Critically low |
| Yepes-Nunez, 2018 | 1 | 2 | 1 | 2 | 1 | 1 | 1 | 2 | 3 | 1 | 2 | 2 | 1 | 1 | 2 | 1 | High |
| Yin, 2019 | 1 | 0 | 1 | 1 | 1 | 1 | 0 | 2 | 3 | 0 | 2 | 2 | 1 | 1 | 2 | 1 | Critically low |
| Yorke, 2015 | 1 | 0 | 0 | 1 | 1 | 1 | 0 | 2 | 1 | 0 | 0 | 0 | 0 | 0 | 0 | 1 | Critically low |
| Zairina, 2014 | 1 | 0 | 0 | 2 | 1 | 1 | 0 | 2 | 1 | 0 | 0 | 0 | 0 | 0 | 0 | 1 | Critically low |
| Zhang, 2019 | 1 | 2 | 0 | 1 | 1 | 1 | 0 | 2 | 3 | 0 | 2 | 2 | 0 | 1 | 1 | 1 | Critically low |
| Zhang, 2021 | 1 | 2 | 0 | 1 | 1 | 1 | 0 | 2 | 3 | 0 | 2 | 1 | 1 | 1 | 2 | 1 | Critically low |
| Zhong, 2017 | 1 | 0 | 1 | 1 | 1 | 1 | 1 | 2 | 3 | 1 | 2 | 2 | 1 | 1 | 1 | 0 | Critically low |
| Zhu, 2022 | 1 | 1 | 0 | 1 | 1 | 1 | 0 | 2 | 3 | 0 | 2 | 1 | 1 | 1 | 1 | 1 | Critically low |
| Zuccotti, 2015 | 1 | 2 | 1 | 1 | 1 | 1 | 0 | 2 | 3 | 0 | 2 | 2 | 1 | 1 | 2 | 1 | Critically low |

**Notes:**

1. Kew KM, Cates CJ. Home telemonitoring and remote feedback between clinic visits for asthma. The Cochrane Database of Systematic Reviews. 2016;2016(8):CD011714.

2. Kew KM, Nashed M, Dulay V, Yorke J. Cognitive behavioural therapy (CBT) for adults and adolescents with asthma. The Cochrane Database of Systematic Reviews. 2016;9(9):CD011818.

3. Kew KM, Malik P, Aniruddhan K, Normansell R. Shared decision-making for people with asthma. The Cochrane Database of Systematic Reviews. 2017;10(10):CD012330.

4. Kew KM, Carr R, Crossingham I. Lay-led and peer support interventions for adolescents with asthma. The Cochrane Database of Systematic Reviews. 2017;4(4):CD012331.

5. Li Q, Zhou Q, Zhang G, Tian X, Li Y, Wang Z, et al. Vitamin D Supplementation and Allergic Diseases during Childhood: A Systematic Review and Meta-Analysis. Nutrients. 2022;14(19).

6. Li X, Mao C, Pan Y. Effect of Routine Therapy Assisted by Physical Exercise on Pulmonary Function in Patients with Asthma in Stable Stage: A Systematic Review and Meta-analysis of Randomized Clinical Trials. Comput Math Methods Med. 2022;2022:2350297.

7. Wang Y, Wang J, Chen L, Zhang H, Yu L, Chi Y, et al. Efficacy of vitamin D supplementation on COPD and asthma control: A systematic review and meta-analysis. Journal of Global Health. 2022;12:04100.

8. Wang Q, Ying Q, Zhu W, Chen J. Vitamin D and asthma occurrence in children: A systematic review and meta-analysis. Journal of Pediatric Nursing. 2022;62:e60-e8.

**^*^ AMSTAR-2 items:**

1. Did the research questions and inclusion criteria for the review include the components of PICO? (Yes: 1/No: 0).

2. Did the report of the review contain an explicit statement that the review methods were established prior to the conduct of the review and did the report justify any significant deviations from the protocol? (Yes: 2/Partial Yes: 1/No: 0).

3. Did the review authors explain their selection of the study designs for inclusion in the review? (Yes: 1/No: 0).

4. Did the review authors use a comprehensive literature search strategy? (Yes: 1/Partial Yes: 1/No: 0).

5. Did the review authors perform study selection in duplicate? (Yes: 1/No: 0).

6. Did the review authors perform data extraction in duplicate? (Yes: 1/No: 0).

7. Did the review authors provide a list of excluded studies and justify the exclusions? (Yes: 1/No: 0).

8. Did the review authors describe the included studies in adequate detail? (Yes: 2/Partial Yes: 1/No: 0).

9. Did the review authors use a satisfactory technique for assessing the risk of bias (RoB) in individual studies that were included in the review? (Yes: 3/Partial Yes: 2/No: 1/Includes only NRSI: 0).

10. Did the review authors report on the sources of funding for the studies included in the review? (Yes: 1/No: 0).

11. If meta-analysis was performed did the review authors use appropriate methods for statistical combination of results? (Yes: 2/No: 1/No meta-analysis Conducted: 0).

12. If meta-analysis was performed, did the review authors assess the potential impact of RoB in individual studies on the results of the meta-analysis or other evidence synthesis? (Yes: 2/No: 1/No meta-analysis Conducted: 0).

13. Did the review authors account for RoB in individual studies when interpreting/ discussing the results of the review? (Yes: 1/No: 0).

14. Did the review authors provide a satisfactory explanation for, and discussion of, any heterogeneity observed in the results of the review? (Yes: 1/No: 0).

15. If they performed quantitative synthesis did the review authors carry out an adequate investigation of publication bias (small study bias) and discuss its likely impact on the results of the review? (Yes: 2/No: 1/No meta-analysis Conducted: 0).

16. Did the review authors report any potential sources of conflict of interest, including any funding they received for conducting the review? (Yes: 1/No: 0).
